# **α**7 Nicotinic acetylcholine receptor mediates right ventricular fibrosis and diastolic dysfunction in pulmonary hypertension

**DOI:** 10.1172/jci.insight.142945

**Published:** 2021-06-22

**Authors:** Alexander Vang, Denielli da Silva Gonçalves Bos, Ana Fernandez-Nicolas, Peng Zhang, Alan R. Morrison, Thomas J. Mancini, Richard T. Clements, Iuliia Polina, Michael W. Cypress, Bong Sook Jhun, Edward Hawrot, Ulrike Mende, Jin O-Uchi, Gaurav Choudhary

**Affiliations:** 1 Vascular Research Laboratory, Providence VA Medical Center, Providence, Rhode Island, USA.; 2 Department of Medicine, Alpert Medical School of Brown University, Providence, Rhode Island, USA.; 3 Biomedical & Pharmaceutical Sciences, University of Rhode Island, Kingston, Rhode Island, USA.; 4 Department of Medicine, University of Minnesota, Minneapolis, Minnesota, USA.; 5 Department of Molecular Pharmacology, Physiology, and Biotechnology, Alpert Medical School of Brown University, Providence, Rhode Island, USA.; 6 Cardiovascular Research Center, Lifespan Cardiovascular Institute, Rhode Island Hospital, Providence, Rhode Island, USA.

**Keywords:** Cardiology, Pulmonology, Fibrosis, Heart failure, Ion channels

## Abstract

Right ventricular (RV) fibrosis is a key feature of maladaptive RV hypertrophy and dysfunction and is associated with poor outcomes in pulmonary hypertension (PH). However, mechanisms and therapeutic strategies to mitigate RV fibrosis remain unrealized. Previously, we identified that cardiac fibroblast α7 nicotinic acetylcholine receptor (α7 nAChR) drives smoking-induced RV fibrosis. Here, we sought to define the role of α7 nAChR in RV dysfunction and fibrosis in the settings of RV pressure overload as seen in PH. We show that RV tissue from PH patients has increased collagen content and ACh expression. Using an experimental rat model of PH, we demonstrate that RV fibrosis and dysfunction are associated with increases in ACh and α7 nAChR expression in the RV but not in the left ventricle (LV). In vitro studies show that α7 nAChR activation leads to an increase in adult ventricular fibroblast proliferation and collagen content mediated by a Ca^2+^/epidermal growth factor receptor (EGFR) signaling mechanism. Pharmacological antagonism of nAChR decreases RV collagen content and improves RV function in the PH model. Furthermore, mice lacking α7 nAChR exhibit improved RV diastolic function and have lower RV collagen content in response to persistently increased RV afterload, compared with WT controls. These finding indicate that enhanced α7 nAChR signaling is an important mechanism underlying RV fibrosis and dysfunction, and targeted inhibition of α7 nAChR is a potentially novel therapeutic strategy in the setting of increased RV afterload.

## Graphical abstract

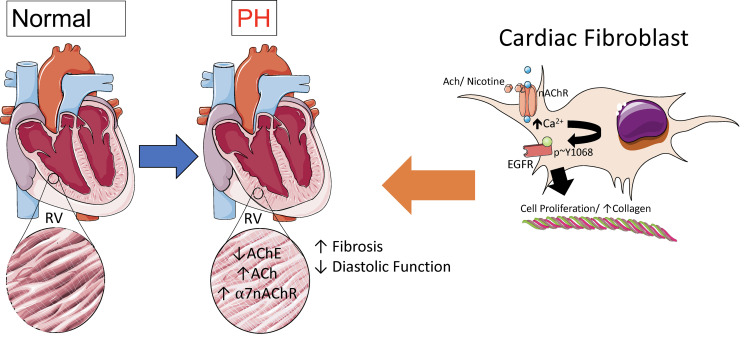

## Introduction

Myocardial fibrosis is a process of pathological extracellular matrix (ECM) remodeling, mediated by activation of cardiac fibroblasts (CFs) (1–3). In response to stress and injury, CFs transform into a more proliferative and hyperactive phenotype, with elevated production of ECM proteins such as collagen (4), resulting in cardiac fibrosis. Mechanisms of increased cardiac fibrosis and fibroblast proliferation/transformation have predominantly been studied in the left ventricle (LV). They include TGF-β signaling, the renin-angiotensin-aldosterone system signaling, adrenergic and endothelin G-protein–coupled receptor signaling, growth factor–mediated tyrosine kinase signaling, and a number of other inflammatory-based pathways (1, 4). Far less is known about the mechanisms driving right ventricular (RV) fibrosis (5–7). This is notable since we and others have demonstrated that some established therapies targeting LV fibrosis, such as angiotensin receptor antagonism, do not attenuate RV fibrosis (8, 9).

RV fibrosis is a key histological feature observed in maladaptive RV hypertrophy and dysfunction. RV dysfunction and failure are associated with poor outcomes in a variety of highly prevalent cardiopulmonary diseases such as heart failure and COPD that are associated with increased RV afterload due to pulmonary hypertension (PH). The poor outcomes related to RV function are most pronounced in pulmonary arterial hypertension (PAH), a disease characterized by progressive pulmonary vascular remodeling resulting in a substantial increase in RV afterload, eventually leading to maladaptive RV hypertrophy, RV dysfunction, and RV failure (10). In preclinical models of PH, RV fibrosis is associated with reduced RV function and cardiac output (11), as well as impaired contractility (12) and stiffness (13) of the RV myocardium. Analogous to animal models, patients with PH and decompensated RV failure have significant RV fibrosis at autopsy (14). Moreover, the extent of RV fibrosis quantified by MRI in patients with PH correlates with both RV ejection fraction and RV end diastolic volume (15). Conversely, little fibrosis is observed in compensated models of RV hypertrophy, despite having similar levels of afterload (11, 14). While RV fibrosis is an important component of decompensated RV in settings of PH, it remains to be determined if mitigating RV fibrosis would improve RV structure and function (6, 16). Moreover, the mechanisms that initiate RV fibrosis and therapeutic targets remain unclear (5, 7).

We have shown that the mechanism that promotes CF proliferation and RV fibrosis related to cigarette smoke exposure is mediated through activation of α7 nicotinic acetylcholine receptor (α7 nAChR) in CF (17). Nonneuronal α7 nAChR has been described in a variety of cell types, and in response to ligand (ACh, nicotine) binding, these pentameric ligand–gated ion channels undergo a conformational change that results in opening of the ion conducting pore (18). The α7 nAChR has a high Ca^2+^ permeability compared with other nAChR isoforms and results in activation of downstream Ca^2+^-dependent signaling pathways (18). Proproliferative effects of α7 nAChR activation have been reported in cancer cells (19–21), vascular smooth muscle cells (22, 23), adventitial fibroblasts (22), and endothelial cells (24, 25), as well as CF (17) — predominantly in settings of nicotine and/or cigarette smoke exposure. Little is known about the proproliferative and profibrotic roles of α7 nAChR in the absence of nicotine exposure. We hypothesized that α7 nAChR on CF mediates RV fibrosis in settings of increased RV afterload. Therefore, in this study, we investigate the role of α7 nAChR and its natural ligand, ACh, in the development of RV fibrosis and dysfunction using human RV tissue, as well as clinically relevant animal models of PH and increased RV afterload. We report that ACh–α7 nAChR signaling is upregulated in the RV in the setting of PH and that antagonism of α7 nAChR results in improved RV fibrosis and function. These findings serve as a conceptual basis for α7 nAChR inhibition as potential therapy for maladaptive RV fibrosis in PH.

## Results

### ACh–α7 nAChR signaling is upregulated in the RV in PH.

**Figure 1:**
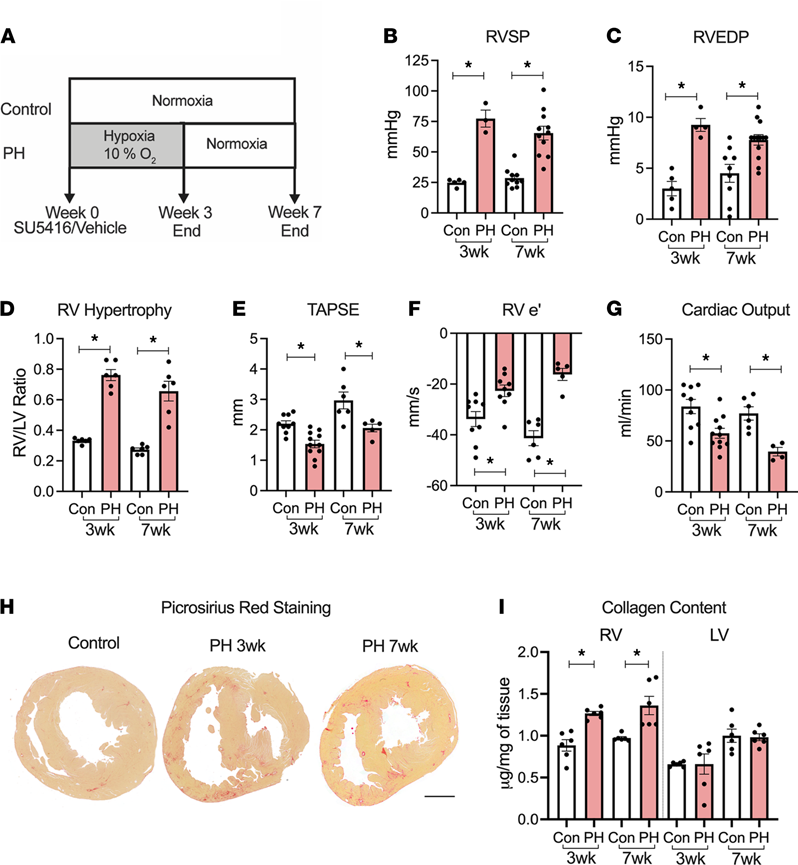
Characterization of pulmonary hypertension model. (**A**) Schematic of the experimental design. (**B** and **C**) Invasively measured RVSP (**B**) and RVEDP (**C**) at 3 weeks (wks) (*n* = 5 control/3 PH) and 7 wks (*n* = 10/11). (**D**) RV weight normalized to the respective LV weight (*n* = 6/6). (**E**–**G**) Echocardiographic measurements at 3-wk (*n* = 9/11) and 7-wk (*n* = 6/5) time points of TAPSE (**E**) using M-Mode, RV free wall early diastolic velocity (e’) using tissue Doppler (**F**), and cardiac output (**G**). (**H**) Representative images of transverse heart sections from control and PH animals stained with Picrosirius red. Scale bar: 5 mm. (**I**) Collagen content measured using hydroxyproline assay from RV and LV homogenates at 3 wks (*n* = 6) and 7 wks (*n* = 6). Two-tailed *t* tests were performed between control and PH at 3wks and 7 wks; *P < 0.05. Con, control; PH, pulmonary hypertension; RV, right ventricle; RVEDP, right ventricular end diastolic pressure; RVSP, right ventricular systolic pressure; RV e’, right ventricle free wall early diastolic velocity (e’); TAPSE, tricuspid annular plane systolic excursion.

In order to assess the role of α7 nAChR signaling in RV fibrosis and dysfunction, we studied a rat PH model at 2 time points (3 and 7 weeks, Figure 1A) that develops severe PH (Figure 1B) with increased RV end diastolic pressure (Figure 1C), RV hypertrophy (Figure 1D), RV systolic and diastolic dysfunction (Figure 1, E and F, respectively), and reduced cardiac output (Figure 1G) in response to SU5416/hypoxia (SuHx) (8, 26). In this PH model, except for an increase in LV ejection fraction at the 7-week time point, no significant morphological and functional changes were observed in the LV, as assessed by echocardiography and hemodynamic measurements, respectively (Supplemental Table 1 and Supplemental Figure 1, A–D; supplemental material available online with this article; https://doi.org/10.1172/jci.insight.142945DS1). Significant fibrotic remodeling in the RV (but not in the LV) was observed concomitant with RV dysfunction in the PH rats in comparison with healthy controls (Figure 1, H and I, and Supplemental Figure 1E). There was no significant effect of sex in RV collagen content at either time point (2-way ANOVA with sex as a source of variation, *P* = 0.70 for the 3-week time point, and *P* = 0.67 for the 7-week time point).

**Figure 2:**
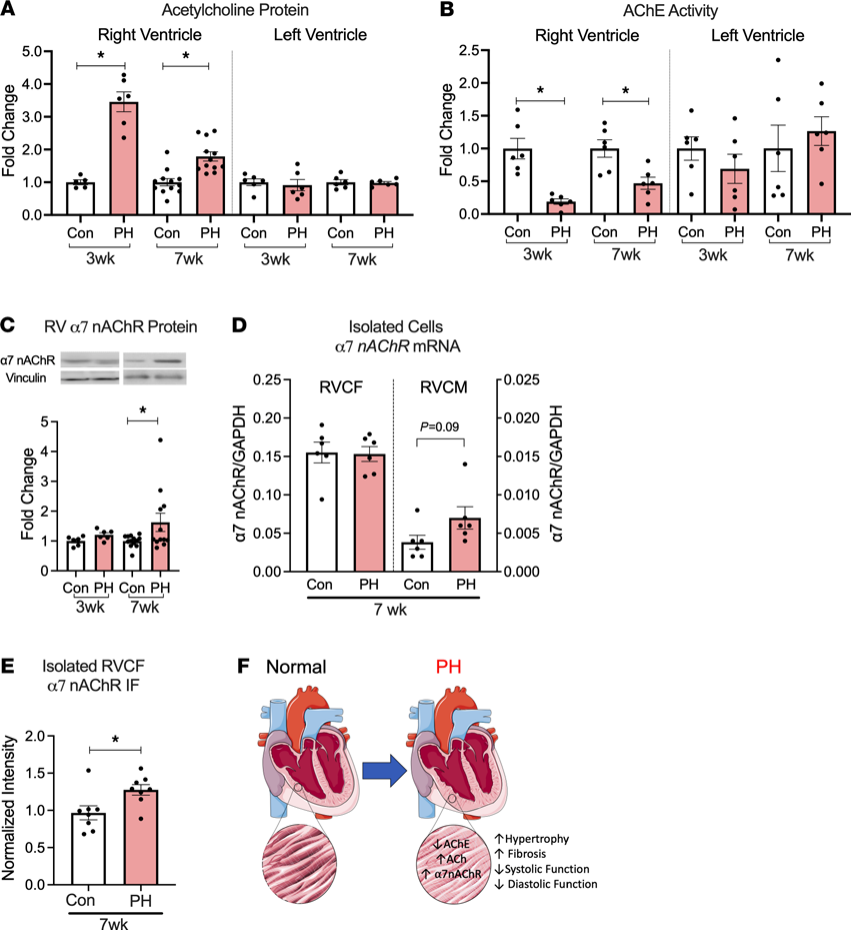
PH is associated with an increase in RV ACh/α7 nAChR. (**A**) ELISA for ACh performed on homogenates from the RV at 3 wks (*n* = 5 Con/6 PH) and 7 wks (*n* = 12 Con/12 PH) and LV at 3 wks and 7 wks (*n* = 6 Con/6 PH). (**B**) AChE activity measured in homogenates from the RV and LV at both time points (*n* = 6 Con/6 PH). (**C**) Expression of α7 nAChR protein relative to Vinculin in RV at 3 wks (*n* = 6 Con/6 PH) and 7 wks (*n* = 13 Con/12 PH) normalized to controls. (**D**) Expression of *α7 nAChR* mRNA in freshly isolated RVCF and RV cardiomyocytes from control and 7 wk PH animals (*n* = 6 Con/6 PH). (**E**) Quantitated immunofluorescence intensity of α7 nAChR in nonpermeabilized isolated RVCF from 7-wk control and PH animals. (**F**) Summary schematic. Data are shown as mean ± SEM. Two-tailed *t* tests were performed between control and PH at 3 and 7 wks; Mann-Whitney *U* test for real-time PCR data. *P < 0.05. ACh, acetylcholine; AChE, acetylcholinesterase; CF, cardiac fibroblasts; CM, cardiomyocytes; Glyceraldehyde 3-phosphate dehydrogenase; IF, immunofluorescence; nAChR, nicotinic acetylcholine receptor; PH, pulmonary hypertension; RV, right ventricle.

We next assessed the changes in ACh signaling in the RV under PH using ventricular myocardium from control and PH animals. ACh levels were increased (Figure 2A) and the activity of ACh degrading enzyme, acetylcholinesterase (AChE), was decreased (Figure 2B) in the RV (but not in the LV) from PH animals compared with controls. Moreover, ACh levels significantly correlated with the fibrosis marker, hydroxyproline content, in the RV (Supplemental Figure 2). We did not find significant changes in expression of ACh synthesis proteins, including vesicular acetylcholine transporter (VAChT), choline acetyltransferase (ChAT), and choline transporter (ChT) (Supplemental Figure 3, A–C). These results suggest that increased ACh content in the RV in PH is predominantly via impaired degradation rather than an increase in ACh synthesis. In addition to increased ACh, the RV from PH rats exhibited increased protein and mRNA expression of α7 nAChR (Figure 2C and Supplemental Figure 3D) at the 7-week time point. There was no significant difference in α7 nAChR expression levels in the LV between control and PH at either time point (Supplemental Figure 3, E and F). While α7 nAChR was predominantly expressed in the CF, overall expression of nAChR remained unaltered in isolated right ventricular cardiac fibroblast (RVCF) from PH rats compared with controls at the 7-week time point (Figure 2D). However, a trend for increased expression was noted in RV cardiomyocytes (Figure 2D). We next assessed the cell membrane expression of α7 nAChR by staining nonpermeabilized RVCF, and we found that RVCF from PH animals had higher cell surface expression of α7 nAChR compared with those isolated from control rats (Figure 2E). In summary, increased RV ACh–α7 nAChR signaling in PH was associated with RV hypertrophy, fibrosis, and dysfunction (Figure 2F).

**Figure 3:**
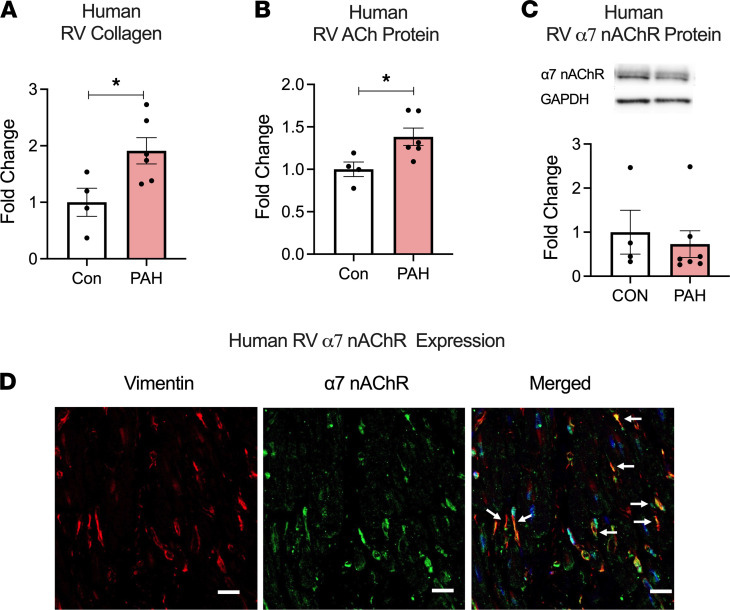
PAH is associated with an increase in RV Collagen and ACh. (**A**–**C**) Collagen (**A**), ACh (**B**), and α7 nAChR (**C**) protein expression assessed using Sircol assay, ELISA, and immunoblot, respectively, in explanted RV tissue from end-stage human PAH (*n* = 5) and controls (*n* = 4). (**D**) Immunofluorescent staining for α7 nAChR (green), vimentin (red), and nuclei (blue) in human RV tissue from control. Arrows demonstrate localization of α7 nAChR in CF (vimentin positive cells). Scale bar: 20 μm. Data are shown as mean ± SEM. Two-tailed *t* tests were performed between control and PAH. *P < 0.05. ACh, acetylcholine; nAChR, nicotinic acetylcholine receptor; PAH, pulmonary arterial hypertension; RV, right ventricle.

In RV myocardium from end-stage human PAH patients (Supplemental Table 2), we found increased expression collagen and ACh (Figure 3, A and B) with no significant differences in expression of α7 nAChR and ACh synthesis proteins (ChT, VAChT, and ChAT) (Figure 3C and Supplemental Figure 4). We did note variability in α7 nAChR protein and mRNA expression in the human RV tissue, likely related to sampling bias and/or underling clinical condition of the patients. Immunofluorescent staining of human RV tissue demonstrated that α7 nAChR is predominantly expressed in CF (Figure 3D).

### ACh induces CF activation via α7 nAChR–mediated EGFR transactivation.

**Figure 4:**
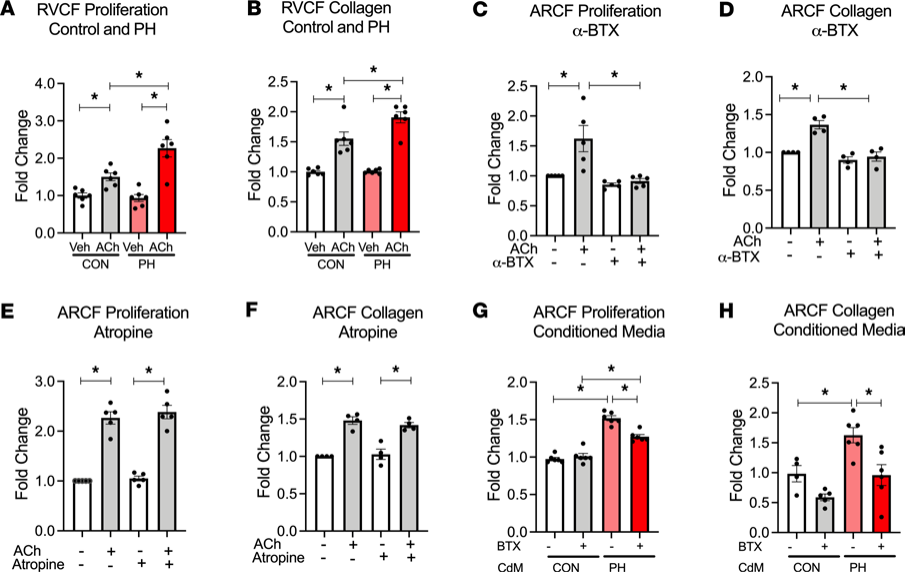
RV cardiomyocyte–derived ACh promotes cardiac fibroblast proliferation and collagen synthesis through α7 nAChR activation. (**A** and **B**) RVCFs isolated from 7-wk control and PH rats treated with vehicle or 10 nM ACh for 24 hours and then assessed for cell proliferation by cell counts (**A**) and collagen content (**B**) with Sircol assay (*n* = 6). (**C** and **D**) Cell counts (*n* = 5) (**C**) and collagen content (*n* = 4) (**D**) of ARCF in response to 10 nM ACh with or without α7 nAChR antagonist α-BTX (100 nM) for 24 hours. (**E** and **F**) Cell counts (*n* = 5) (**E**) and collagen content (*n* = 4) (**F**) of ARCF in response to 10 nM ACh in the presence or absence of muscarinic receptor antagonist atropine (50 μM) for 24 hours. (**G** and **H**) Cell count (**G**) and collagen content (**H**) from ARCFs treated with conditioned media from isolated 7 wks Con/PH RV cardiomyocytes. Data are shown as mean ± SEM. ANOVA followed by Bonferroni comparison. *P < 0.05. ARCF, Adult rat cardiac fibroblasts; RVCF, right ventricular cardiac fibroblasts; ACh, acetylcholine; α-BTX, α-bungarotoxin; RVCM, right ventricular cardiac myocytes; CdM, conditioned media (from RV cardiomyocytes); CON, control; PH, pulmonary hypertension.

We hypothesized that upregulation of ACh–α7 nAChR signaling contributes to CF activation in the RV in PH. We stimulated primary RVCF isolated from control and PH rats (7-week time point) with ACh, a natural ligand for α7 nAChR, and we found that ACh significantly increased CF proliferation and collagen content (Figure 4, A and B) compared with vehicle treatment. Moreover, the relative increases in ACh-mediated CF proliferation and collagen content were higher in CF from PH compared with control rats (Figure 4, A and B). The effect of ACh on CF proliferation and collagen content were blocked by an α7 nAChR–specific antagonist α-bungarotoxin (α-BTX) (Figure 4, C and D), or a nonselective and noncompetitive nAChR antagonist mecamylamine (Mec, Supplemental Figure 5A), but not by a muscarinic receptor antagonist atropine (Figure 4, E and F, and Supplemental Figure 5B), demonstrating that the ACh effect on CF proliferation is mediated via α7 nAChR. Similar to ACh, conditioned media from cardiomyocytes isolated from RVs of PH animals (7-week time point) induced a significant increase in both CF proliferation and collagen content compared with media from cardiomyocytes isolated from control animals (Figure 4, G and H). These changes induced by conditioned media were attenuated in the presence of α-BTX, suggesting that cardiomyocyte-derived ACh results in CF proliferation and fibrosis in RV in settings of PH.

**Figure 5:**
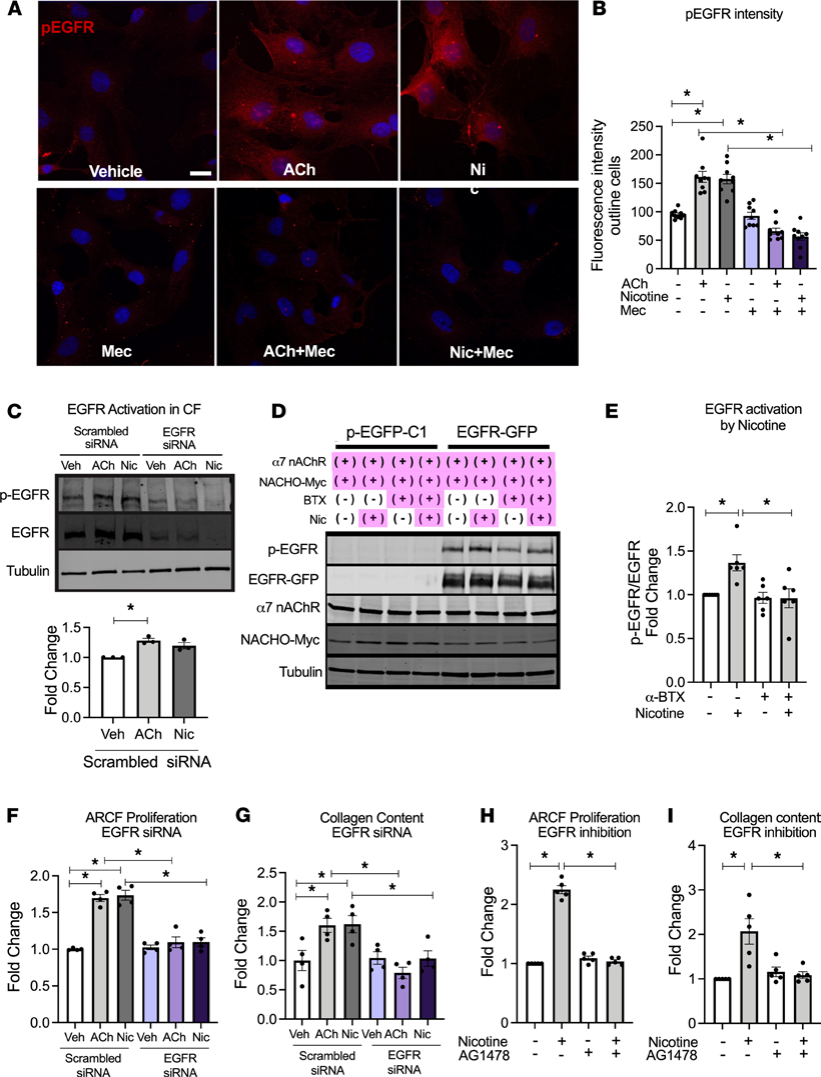
Cardiac fibroblast proliferation and collagen synthesis are mediated via α7 nAChR–EGFR transactivation. (**A**) Representative fluorescence image of containing p-EGFR (Y^1068^) (red) and nuclei (blue by Hoechst**)** in ARCF 30 minutes after vehicle, ACh (10 nM), or nicotine (600 nM) treatment in the presence or absence of Mec (20 μM). Scale bar: 20 μm. (**B**) Summary data from **A** (*n* = 9). (**C**) EGFR transactivation in ARCFs treated with vehicle, 600 nM nicotine, or 10 nM ACh for 30 minutes. EGFR activation was estimated by p-EGFR(Y^1068^)/EGFR (*n* = 3). EGFR siRNA and scrambled siRNA-transfected cells were used to confirm the antibody specificities. (**D**) EGFR transactivation in HEK293T-overexpressing EGFR, α7 nAChR, and Myc-tagged NACHO, by nicotine (600 nM, 30 min) in the presence or absence of α-BTX. Cells stably overexpressing EGFP were used as a control. EGFR phosphorylation, overexpressed EGFR, α7 nAChR, and NACHO were detected by the antibodies against p-EGFR (Y^1068^), GFP, α7 nAChR, and Myc, respectively. (**E**) Summary data from **D** (*n* = 6). p-EGFR/EGFR was normalized to that in cells without nicotine and α-BTX treatment. (**F** and **G**) Cell counts (*n* = 4) (**F**) and collagen content (*n* = 4) (**G**) of ARCF in response to ACh (10 nM) or nicotine (600 nM) in cells transfected with either EGFR specific or scrambled siRNA. (**H** and **I**) Cell counts (*n* = 5) (**H**) and collagen content (*n* = 5) (**I**) of ARCF in response to nicotine (600 nM) in presence or absence of EGFR inhibitor AG1478 (50 μM). Data are shown as mean ± SEM. ANOVA followed by Bonferroni comparison. *P < 0.05. ACh, acetylcholine; α-BTX, α-bungarotoxin; AG1478, EGFR inhibitor; Mec, mecamylamine; nAChR, nicotinic acetylcholine receptor.

We next investigated the mechanism of how α7 nAChR–mediated signaling promotes CF activation. Some reports have suggested potential interactions between α7 nAChR signaling and epidermal growth factor receptor (EGFR) signaling (27). EGFR plays an important role in cell survival and proliferation in a number of cell types, including CF (28). Therefore, we investigated if activation of nAChR would lead to EGFR activation in CF. Using IHC, we found that ACh or nicotine stimulation increased EGFR phosphorylation in adult CF, and this phosphorylation was significantly inhibited in the presence of a nAChR antagonist, Mec (Figure 5, A and B). Ach- and nicotine-mediated EGFR phosphorylation was also confirmed by Western blotting of protein lysates from the CF (Figure 5C). A similar result regarding EGFR phosphorylation and its inhibition was confirmed by Western blot in HEK-293T cells overexpressing EGFR, α7 nAChR, and its chaperone protein TMEM35/NACHO (Supplemental Figure 6) (29) using α-BTX (Figure 5, D and E). In contrast, activation of α7 nAChR was not associated with phosphorylation of another tyrosine kinase growth factor receptor, fibroblast growth factor receptor (FGFR, Supplemental Figure 7). Moreover, ACh- and nicotine-induced CF proliferation and collagen synthesis were abolished by knocking down of EGFR using siRNA (Figure 5, F and G, and Supplemental Figure 8), or pretreatment with selective EGFR inhibitor AG1478 (Figure 5, H and I, and Supplemental Figure 9). Together, these results suggest that α7 nAChR–mediated CF activation is dependent on EGFR transactivation.

**Figure 6:**
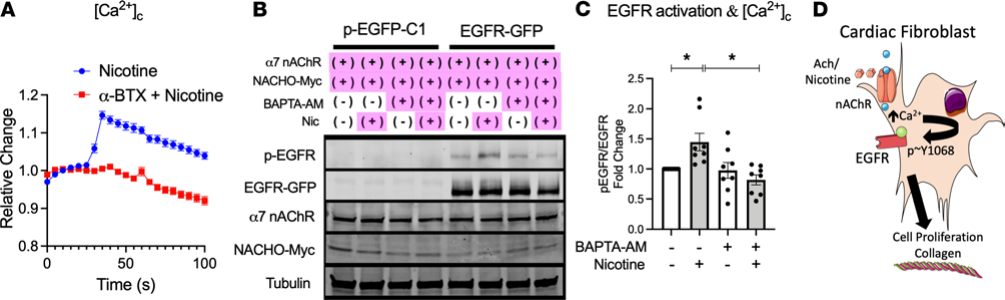
Activation of α7 nAChR results in Ca^2+^-mediated EGFR transactivation. (**A**) Averaged time course of the changes in [Ca^2+^]_c_ in ARCF in response to 600 nM nicotine treatment (added at 30 seconds) in the presence (red squares, *n* = 63) or absence of 100 nM α-BTX (blue circles, *n* = 34). Data normalized to before nicotine stimulation. (**B**) EGFR transactivation in HEK293T overexpressing EGFR, α7 nAChR, and NACHO in presence of a cell-permeable Ca^2+^ chelator BAPTA-AM. (**C**) Summary data from **B** (*n* = 8). (**D**) Proposed mechanism of α7 nAChR–activated proliferation and enhanced collagen content in CF. Data are shown as mean ± SEM. ANOVA followed by Bonferroni comparison. *P < 0.05. ACh, acetylcholine; α-BTX, α-bungarotoxin; nAChR, nicotinic acetylcholine receptor; BAPTA, 1;2-bis(o-aminophenoxy)ethane-N;N;N′;N′-tetraacetic acid.

Since homomeric α7 nAChR proteins form ligand-gated cation (e.g., Ca^2+^) channels at the plasma membrane (30), we performed live cell imaging with cell-permeable Ca^2+^-sensitive dyes to confirm the functional expression of α7 nAChR. We demonstrate a transient increase of cytosolic Ca^2+^ concentration ([Ca^2+^]_c_) in response to the nAChR-specific agonist, nicotine, in adult rat CFs that was abolished in the presence of the α7 nAChR blocker α-BTX (Figure 6A and Supplemental Figure 10). α7 nAChR–mediated Ca^2+^ mobilization serves as an important second messenger in other cell types. Therefore, we tested if cytosolic Ca^2+^ elevation is mechanistically involved in α7 nAChR–dependent EGFR transactivation. We found that pretreatment with the cell-permeable Ca^2+^ chelator BAPTA-AM significantly inhibited α7 nAChR stimulation–mediated EGFR phosphorylation (Figure 6, B and C).

In summary, our data demonstrate that ACh stimulation promotes CF proliferation via α7 nAChR and Ca^2+^-mediated EGFR transactivation in primary adult CFs (Figure 6D), and this is likely the underlying mechanism by which increased ACh results in cardiac fibrosis in the RV in the setting of PH in vivo.

### Inhibition of nAChR improves RV function in experimental PH.

**Figure 7:**
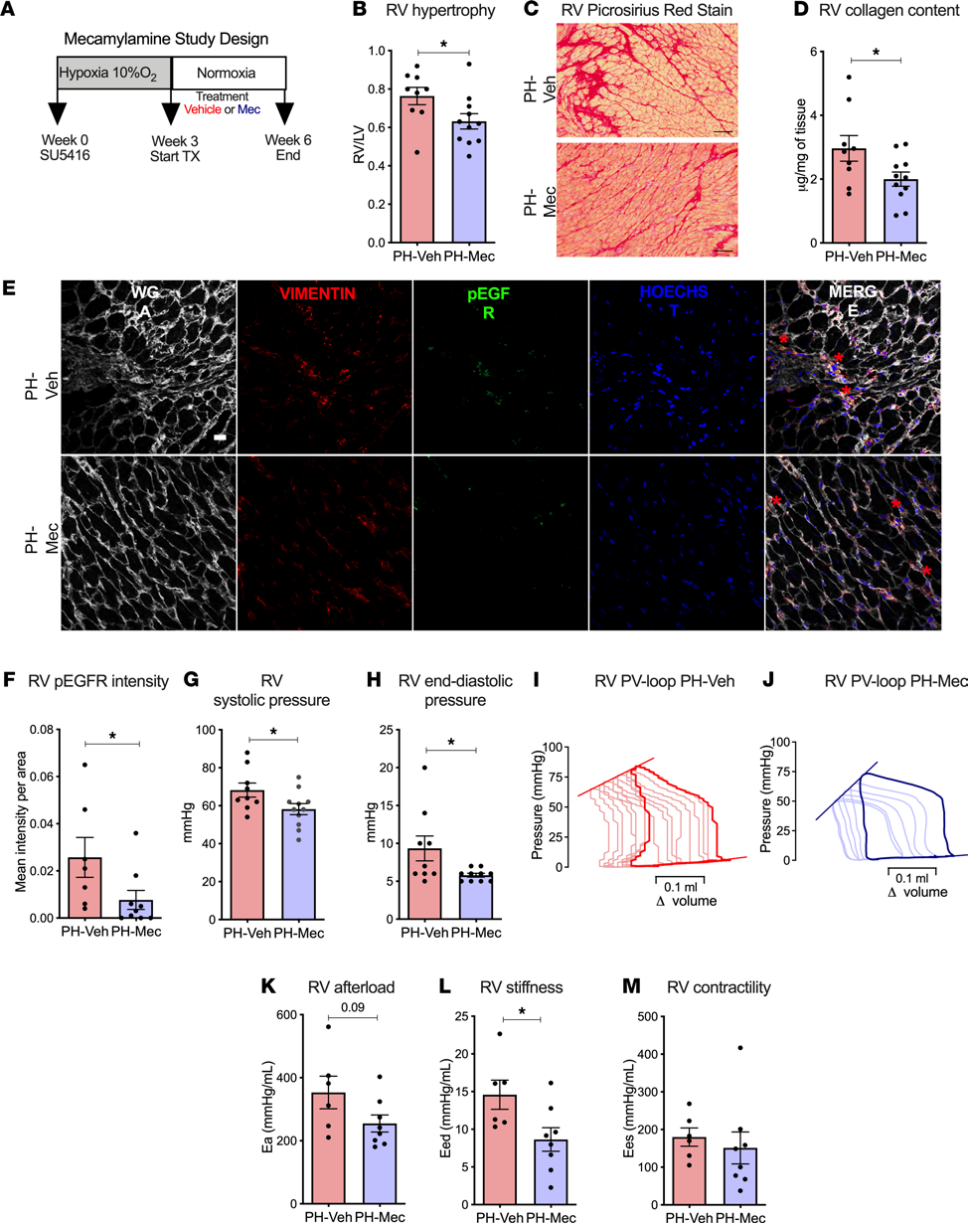
Treatment with nAChR blocker mecamylamine improves RV function in experimental pulmonary hypertension. (**A**) Study design for mecamylamine (Mec) treatment. PH rats were randomized into vehicle (PH-Veh) or Mec (20 mg/kg, i.p., PH-Mec) treatment daily for 3 wks (*n* = 9 PH-Veh/11 PH-Mec). (**B**) RV weight normalized to the respective LV weight (*n* = 9 PH-Veh/11 PH-Mec). (**C**) Representative images of the RV from vehicle- and Mec-treated animal stained with Picrosirius red. Scale bar: 20 μm. (**D**) Collagen content in RV homogenates from vehicle- and Mec-treated PH rats (*n* = 7 PH-Veh/9 PH-Mec). (**E**) Representative immunofluorescence staining for pEGFR (Y^1068^) in the RV from vehicle- and Mec-treated rats. Wheat germ agglutinin to outline the cardiomyocytes in white (Alexa 647), vimentin for cardiac fibroblasts in red (Alexa 594), pEGFR in green (Alexa488), and Hoechst for nuclei in blue. Red asterisks show colocalization of pEGFR with fibroblasts. Scale bar: 20 μm. (**F**) Quantification of area of pEGFR immunofluorescence in the RV (*n* = 7 PH-Veh/9 PH-Mec). (**G** and **H**) Invasively measured RV systolic pressures (*n* = 9 PH-Veh/11 PH-Mec) (**G**) and RV end diastolic pressures (*n* = 9 PH-Veh/10 PH-Mec) (**H**). (**I** and **J**) Representative examples of pressure-volume relationship for vehicle- (**I**) and Mec-treated (**J**) PH rats. (**K**–**M**) Arterial Elastance (Ea) as a measure of RV afterload (*n* = 6 PH-Veh/8 PH-Mec) (**K**), end-diastolic pressure-volume relationship (Eed) as an indicator of RV stiffness (*n* = 6 PH-Veh/8 PH-Mec) (**L**), and end-systolic pressure-volume relationship (Ees) as a measure of RV contractility (*n* = 6 PH-Veh/8 PH-Mec) (**M**) derived from the PV loop analyses. Data are shown as mean ± SEM. Two-tailed unpaired *t* tests or Mann-Whitney *U* test between vehicle and Mec treatment. *P < 0.05. Ea, arterial elastance; Eed, end-diastolic pressure-volume relationship; Ees, end-systolic pressure-volume relationship; LV, left ventricle; Mec, mecamylamine; PH, pulmonary hypertension; RV, right ventricle; RVSP, RV systolic pressure; RVEDP, RV end diastolic pressure; or RV contractility; WGA, wheat germ agglutinin.

Since our in vitro data strongly suggest a significant role of ACh–α7 nAChR signaling in causing CF proliferation and collagen synthesis, we next investigated the role of ACh–α7 nAChR signaling in PH-induced RV dysfunction in vivo by treating PH rats (SuHx model) with nAChR antagonist Mec (20 mg/kg/d i.p. for 3 weeks, Figure 7A). Mec was administered after the establishment of PH, which was confirmed by echocardiogram (Supplemental Table 3). We found that Mec treatment significantly reduced RV hypertrophy (Figure 7B), RV collagen content (Figure 7, C and D), and EGFR phosphorylation (Figure 7, E and F) in comparison with the vehicle-treated group. These RV changes were associated with significantly improved stroke volume and RV diastolic function (RV e’) as assessed by echocardiography (Supplemental Table 3) and significantly reduced RV systolic and end diastolic pressures in the Mec-treated group compared with the vehicle group (Figure 7, G and H). In order to assess load-independent parameters of RV function, RV pressure-volume analysis (Figure 7, I and J) was performed. We found that treatment with Mec resulted in a trend toward RV afterload reduction (Figure 7K) and significantly reduced RV end diastolic stiffness (Figure 7L), without a significant effect in RV contractility (Figure 7M). There were no changes in tail blood pressure, LV pressures (Supplemental Figure 11), or LV systolic function (Supplemental Table 3). Lung histology demonstrated a trend toward decrease in vascular medial thickness in Mec-treated PH rats compared with vehicle-treated PH rats, while the levels of vessel occlusion were not altered (Supplemental Figure 12). Taken together, these findings support our hypothesis that nAChR signaling plays an important role in causing RV fibrosis and dysfunction, and that pharmacologically blocking nAChR can be a potential therapeutic option to mitigate RV fibrosis and improve RV diastolic function in PH. In order to assess if Mec inhibition remains effective in attenuating RV fibrosis and collagen content in a more advanced stage of PH, we performed another treatment experiment. In this experiment we started Mec administration at the 5-week time point instead of the 3-week time point and administered the drug for 3 weeks (Supplemental Figure 13). We found that later administration of Mec resulted in similar findings, along with reduction in RV collagen and improvement of RV diastolic function (Supplemental Figure 13 and Supplemental Table 4).

### RV pressure overload induces RV fibrosis via α7 nAChR.

**Figure 8:**
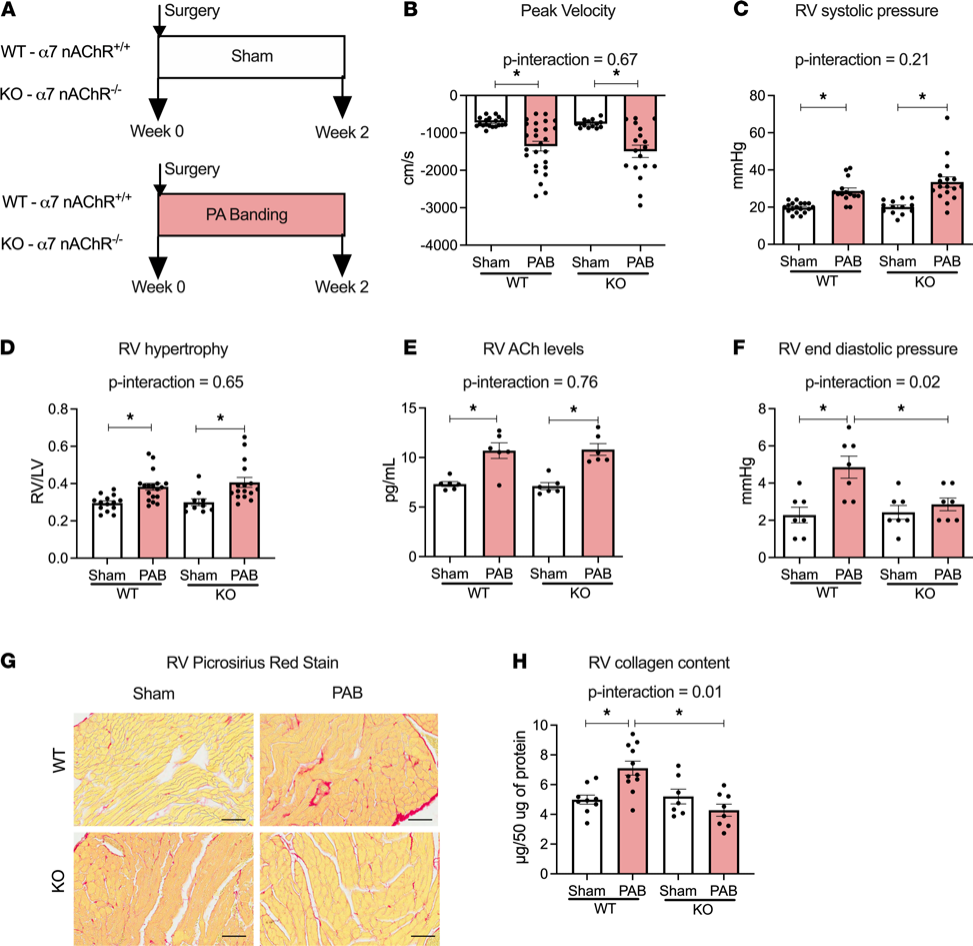
RV collagen content is dependent on α7 nAChR in RV pressure overload. (**A**) Study design of PAB model with fixed RV afterload; WT and α7 nAChR–KO mice were randomized to PAB or sham surgery and followed for 2 wks. (**B** and **C**) Gradient across the banding site (*n* = 14–18) assessed by echocardiography (**B**) and RV systolic pressure assessed by invasive hemodynamics (*n* = 13–18) (**C**). (**D**) RV weight normalized to respective LV weight (*n* = 10–18). (**E**) ACh levels in RV homogenates (*n* = 6). (**F**) RV end diastolic pressure. (**G**) Representative images of RV from WT and KO mice after sham or PA banding surgery stained with Picrosirius red. Scale bar: 20 μm. (**H**) RV collagen content by Sircol assay (*n* = 7–11). Data are shown as mean ± SEM. Two-way ANOVA followed by Bonferroni comparison test. *P < 0.05. PAB, pulmonary artery banding; RVSP, RV systolic pressure; RVEDP, RV end diastolic pressure.

In order to further delineate that RV fibrosis in PH is mediated via α7 nAChR in the heart, we utilized α7 nAChR–KO mice (α7nAChR^–/–^, Supplemental Figure 14) subjected to fixed RV afterload by pulmonary artery banding (PAB) (Figure 8A). The KO mice grow to normal size and showed no obvious physical or cardiac deficits (31) (Supplemental Table 5). Both WT (α7nAChR^+/+^) and KO mice subjected to PAB showed a similar pressure gradient across the banding site and a similar increase in RV systolic pressure (RVSP) compared with sham-operated animals (Supplemental Table 5 and Figure 8, B and C). Although both WT and KO mice after PAB developed similar levels of RV hypertrophy, afterload, and increased ACh content (Figure 8, D and E), significant RV diastolic dysfunction and fibrosis were only observed in WT mice (Figure 8, F–H) but not in the KO mice when compared with sham counterparts. There were no significant changes in LV pressures, LV morphology and function among all groups (Supplemental Table 5 and Supplemental Figure 15). This set of experiments clearly demonstrates that α7 nAChR in the heart mediates RV fibrosis and dysfunction in the setting of increased RV afterload.

## Discussion

RV fibrosis, dysfunction, and failure due to persistently increased afterload is the predominant cause of morbidity and mortality in patients with PH, and defining mechanisms leading to RV dysfunction that may be targeted therapeutically represents a critical unmet need in the field. Patients with PH demonstrate increased RV collagen deposition and increased RV expression of ACh. Using experimental models of PH and increased RV afterload, we demonstrate here that increased RV afterload is not only associated with increased RV ACh–α7 nAChR signaling, but that increased CF proliferation and collagen content depend on the activation of the α7 nAChR/Ca^2+^/EGFR signaling axis. Moreover, we demonstrate that both therapeutic inhibition of α7 nAChR during PH and lack of the α7 nAChR gene in the setting of stable increased RV afterload reduces RV collagen content and consequently improves RV diastolic function. Therefore, targeted inhibition of α7 nAChR as a therapeutic strategy may provide a novel approach to improve the adverse RV remodeling associated with increased morbidity and mortality in patients with PH.

LV hypertrophy and failure have been associated with an increase in cholinergic transdifferentiation (32) and an induction of ACh production by cardiomyocytes in order to preserve homeostasis of cardiac function through cardiomyocyte muscarinic receptors (33, 34). We demonstrate here that RV ACh levels are increased in the setting of RV afterload. The ACh in the RV is partly derived from RV cardiomyocytes in PH, as demonstrated by our experiments using conditioned media from cardiomyocytes. The mRNA expression of key ACh synthesis proteins did not change in PH, but the AChE activity in the RV was consistently lower in PAH patients and in PH animals compared with respective controls. These observations indicate that the increase in ACh is likely through decreased degradation via AChE rather than ACh synthesis. The underlying mechanisms regulating AChE activity in RV in settings of PH remains unclear and needs further investigation. Nonetheless, our results are consistent with prior data, showing decreases in RV AChE both in RV tissue from PAH patients and in a preclinical model of PH (35). It was reported that increasing ACh levels by further inhibition of AChE was associated with restoration of autonomic function, salutary effects on pulmonary vascular remodeling, and resulting improvement in RV function (35). In contrast, our data demonstrate increased ACh significantly correlates with the RV fibrosis marker and may also adversely contribute to increased RV collagen and diastolic dysfunction by activation of CF α7 nAChR.

α7 nAChR is a ligand-gated ion channel expressed in a number of nonneuronal tissues, including CF, and upon activation, it can result in CF proliferation and collagen production (17). The expression of α7 nAChR is increased in the RV in experimental PH. At a cellular level, cardiomyocytes have substantially lower expression of α7 nAChR compared with CF both in control and PH, but they show a trend toward increased expression in PH. In contrast, isolated RVCF from PH did not show an increase in α7 nAChR mRNA expression, suggesting that the observed increase in α7 nAChR in the whole RV lysate was likely as a result of an increase in CF number and an increase in cardiomyocyte α7 nAChR expression. Despite unchanged α7 nAChR transcript levels, RVCF from PH animals had higher proliferation and collagen production in response to ACh. This was likely a result of increased expression of α7 nAChR at the plasma membrane of CF and/or increased protein stability of α7 nAChR in the CF. It is known that protein expression and membrane trafficking of nAChR can be regulated by ligand presence, a number of chaperone proteins, and alteration in proteasomal degradation (36). Further studies are needed to elucidate the underlying mechanism of increase in α7 nAChR protein, despite similar transcript in PH CF.

As mentioned, α7 nAChR has high Ca^2+^ permeability relative to other nAChR isoforms. We demonstrate that α7 nAChR activation in CF results in an increase in intracellular Ca^2+^. We further show that α7 nAChR activation by its ligands can transactivate EGFR and subsequently increases CF proliferation and collagen production. In vivo, inhibition of nAChR resulted in a decrease in EGFR phosphorylation in the RVCF in experimental PH. These data support an important role of EGFR transactivation in mediating the effects of nAChR activation. EGFR can be activated by binding to its ligand or intracellularly via transactivation by various signaling proteins, as well as posttranslational modifications including phosphorylation. While it is well known that transactivation of EGFR by G-protein–coupled receptors can activate CF and induce cardiac fibrosis (28, 37, 38), to the best of our knowledge, nAChR-mediated transactivation of EGFR in CF has not been previously reported. Since pretreatment of [Ca^2+^]_c_ chelator BAPTA-AM eliminated the effects of α7 nAChR activation on EGFR, [Ca^2+^]_c_ mobilization via ligand-mediated α7 nAChR opening appears to be required for EGFR transactivation via the α7 nAChR. This mechanism is consistent with prior data on the involvement of Ca^2+^-dependent kinases or calmodulin-dependent kinases in the mechanism of EGFR activation (39–41). However, further studies are required to delineate the precise signaling mechanism of α7 nAChR–mediated EGFR transactivation in CF and identify the specific Ca^2+^-dependent molecules participating in this pathway.

The significance of cardiomyocyte α7 nAChR expression and whether it is adaptive or maladaptive needs to be investigated in future studies. Inhibition or gene deletion of α7 nAChR did not affect RV systolic function or RV mass, suggesting that α7 nAChR does not mediate the potential beneficial effects of ACh on RV systolic function. Interestingly, we observed that, in addition to the effects on RV collagen, nAChR inhibition was associated with decreased RVSP and reduced RV hypertrophy. These changes were associated with a trend in decreased vascular remodeling in the lungs. It has been shown that activation of α7 nAChR results in proliferation of in vascular smooth muscle cells, vascular adventitial fibroblasts (22), and endothelial cells (42), and an increase in PA pressure (43). It is likely that these vascular effects led to reductions in RVSP upon treatment with nAChR antagonist, potentially confounding effects on RV remodeling. To overcome this concern, we used the PA banding model of fixed RV afterload to confirm that lack of α7 nAChR was still associated with both decreased collagen content and improved diastolic function in the RV, despite having similar RV mass. Collectively, the data support that, in setting of increased RV afterload, ACh in the RV plays both a homeostatic role by improving cardiomyocyte function and reducing inflammation (35), and a maladaptive role by increasing CF proliferation and collagen production via α7 nAChR stimulation in CF. Therefore, therapeutic approaches targeting the maladaptive receptor signaling rather than targeting the ligand may have a more beneficial impact.

While PAH is rare, PH is a prevalent condition associated with several cardiopulmonary diseases. Up to half of all echocardiograms and over 80% of patients undergoing right heart catheterizations reveal elevated pulmonary artery pressure (PAP) (44–47). Even mildly elevated PAP can be associated with RV dysfunction (48). The presence of RV dysfunction is consistently associated with poor prognosis (49). In addition to RV systolic function, the presence of RV diastolic stiffness (diastolic dysfunction) is also related to clinical PAH progression at both baseline and follow-up after treatment (50). Furthermore, RV diastolic stiffness corrected for RV wall thickness was increased in patients with poor prognosis (50), suggesting that factors independent of RV hypertrophy play a role in the relationship of RV diastolic function and outcomes. RV fibrosis is associated with maladaptive RV remodeling in patients and preclinical animal models, and it is related to impaired RV diastolic stiffness (11, 14).

While RV fibrosis and function improve by reducing in PA pressures, therapeutic strategies to improve RV diastolic function and RV fibrosis in the context of fixed RV afterload are limited (5). This is particularly important because, despite increasing availability of therapies for PAH, the average improvement in mean PA pressures with treatment remain modest (51). Preclinical studies have identified some therapeutic strategies like beta blockers (52), iloprost (53), and inhibitors of p38 MAPK signaling (54, 55) that have reduced RV fibrosis and improved RV function despite persistent increase in afterload. In contrast, treatment with pirfenidone or galectin-3 inhibitor reduced RV fibrosis without improving RV function (56), suggesting heterogeneity between antifibrotic therapies. It has also been reported that, in PAH, both myofibril- and fibrosis-mediated stiffness may contribute to increased RV myocardial stiffness (13, 57). While we did not evaluate myofibril properties in the current study, we have previously shown that nicotine had no effect on contractile properties of isolated primary cardiomyocytes (17). Therefore, the nAChR-mediated diastolic dysfunction is likely mediated though the receptors on the CF and consequent fibrosis.

Our study introduces inhibition of α7 nAChR signaling as a potentially novel approach to mitigate maladaptive RV fibrosis and improve RV diastolic function. However, additional studies will be required to identify the most effective and safe α7 nAChR blocker and investigate the potential clinical significance of targeting this pathway in patients to improve symptoms and outcomes related to RV dysfunction.

In conclusion, we demonstrate that CF α7 nAChR signaling plays an important role in mediating RV fibrosis and dysfunction in settings of elevated RV afterload and may be therapeutically targeted to improve RV fibrosis and function in patients with PH.

## Methods

### Materials

#### Reagents.

All chemicals and reagents used were purchased from Sigma-Aldrich except α-BTX (Abcam), Lipofectamine3000 and Lipofectamine RNAiMAX (Thermo Fisher Scientific), Collagenase II and Deoxyribonuclease (Worthington Biochemicals Corporation), BAPTA-AM (Invitrogen and Cayman Chemical), and heparin (MWI Animal Health). A pool of three 19–25 nt siRNAs against rat EGFR (sc-108050) and the control siRNA (sc-37007) were purchased from Santa Cruz Biotechnology Inc.

#### Antibodies.

Antibodies used are shown in Supplemental Table 6.

#### Plasmids.

Human α7 nAChR in pcDNA3.1 (a gift from Sherry Leonard [University of Colorado Denver, Denver, Colorado, USA] and Henry Lester [California Institute of Technology, Pasadena, California, USA]; Addgene plasmid 62276; http://n2t.net/addgene:62276; RRID: Addgene_62276) (58), GFP-tagged EGFR in pEGFP-N1 (a gift from Alexander Sorkin [University of Pittsburgh, Pittsburgh, Pennsylvania, USA]; Addgene plasmid 32751; http://n2t.net/addgene:32751; RRID: Addgene_32751) (59); flag-tagged FGFR1 in pWZL Neo Myr (from William Hahn [Dana Farber Cancer Institute] and Jean Zhao [Harvard University, Cambridge, Massachusetts, USA], Addgene plasmid 20486; http://n2t.net/addgene:20486: RRID: Addgene_20486) (60); pWZL-Neo-Myr-Flag-DEST (from Jean Zhao, Addgene plasmid 15300: http://n2t.net/addgene:15300; RRID: Addgene_15300) (60); pcDNA3.1(+) (Invitrogen); PEGFP-C1 (Clontech); and Myc-tagged human transmembrane protein 35 (TMEM35/NACHO) in pLV[Exp]-mCherry-NSE (provided by Phu Tran at University of Minnesota, Minneapolis, Minnesota, USA).

#### Human tissue.

Autopsy human RV samples were obtained from Cleveland Clinic, and 2 control subjects were obtained from BioChain Institute. See Supplemental Table 2 for more information.

### Animal models

#### Rat model of PH.

Male and female adult Sprague-Dawley rats (150–175 g, strain no, 001) were purchased from Charles River Laboratories. PH was induced as previously described (8) by a single s.c. injection of a vascular endothelial growth factor inhibitor (SU5416, 20 mg/kg body mass; Cayman Chemical) that was dissolved in a diluent containing 0.5% carboxymethylcellulose, 0.9% NaCl, 0.4% polysorbate 80, and 0.9% benzyl alcohol (MilliporeSigma), followed by 3 weeks of normobaric hypoxia exposure (10% FiO_2_; Biospherix Ltd.) and subsequently housing them at normoxic conditions for additional 4 weeks. The control group received a diluent injection and was housed in normoxic conditions until the end of study (Figure 1A). At the end of weeks 3 and 7, the animals underwent a transthoracic echocardiogram, followed by invasive hemodynamics, and were then euthanized under isoflurane by exsanguination for tissue collection.

#### α7 NAChR-KO mice model and PAB.

*Chrna7*-null mice (α7 nAChR^–/–^) bred into the C57BL/6J background and originally derived by the laboratory of Arthur Beaudet (Baylor College of Medicine, Baylor, Texas, USA) (31), were obtained from The Jackson Laboratory (stock no. 003232). The mice were subsequently maintained and bred at the Providence VA Medical Center Veterinarian Medical Unit. The genetic background of the mice was confirmed by PCR using the following primers (Integrated DNA Technologies): common primer (5′-TTCCTGGTCCTGCTGTGTTA-3′), WT primer (390 bp) (5-ATCAGATGTTG-CTGGCATGA-3′), and mutant primer (187 bp) (5′-CCCTTTATAGATTCGCCC TTG-3′) as described by The Jackson Laboratory. C57BL/6J mice were used as experimental controls. PAB was used as a RV pressure overload model. Six- to 8-week-old male and female mice were anesthetized and subsequently intubated (BioTex Research), ventilated at a constant ventilator pressure of 13–15 mmHg with 1.5%–2% isoflurane with balanced medical oxygen and PEEP at 2 cm of H_2_O (MiniVent, Harvard Apparatus). A thoracotomy was performed between the second and third intercostal space to access the pulmonary artery (PA). A titanium clip (Teleflex) was applied to create an occlusion of 0.6 mm resulting in a 50% reduction in luminal diameter as previously described (61). The thoracic cavity was sutured, and the animal was allowed to recover from anesthesia before being placed back in its cage. Postoperative analgesia was achieved with buprenorphine (0.1 mg/kg, s.c., b.i.d.) for at least 72 hours. For sham controls, animals underwent the same procedure without placing the 0.6 mm titanium clip around the PA trunk. After 2 weeks, animals underwent a transthoracic echocardiogram and invasive hemodynamics, and they were then euthanized under isoflurane by exsanguination for tissue collection. Some animals from week 7 were used for the isolation of primary RV cardiomyocytes and RVCF.

#### Mec treatment.

Three-week and 5-week PH rats were randomized into vehicle (DMSO) or Mec treatment (20 mg/kg/d, i.p.) groups. After 3 weeks of drug administration, animals underwent terminal procedures before being euthanized.

#### Tissue collection.

Animals were euthanized via exsanguination under anesthesia. Heart and lungs were removed. The heart was separated into RV, LV, and septum; it was weighed and flash frozen in liquid nitrogen. In some animals, the heart was embedded in OCT for cryosectioning. The pulmonary vasculature was perfused via the PA with saline to flush out blood in the lungs. The right side of the lungs was flash frozen in liquid nitrogen, and the left side was fixed in 10% neutral-buffered formalin at 20 cm H_2_O, followed by submersion overnight. Tissues were then paraffin embedded, sectioned (5 μm sections), and stained.

The animals were randomly selected for different assays. The differences in *n* reflect the different processing of the tissue samples for the assays, as well as availability and integrity of tissue due to length of storage. There were some missing data in the echocardiograms because not all parameters could be reliably collected in all animals due to technical reasons in performing echocardiography. Separate individuals performed the echocardiograms and performed the measurements and analyses of the echocardiograms. Both were blinded to experimental condition.

### In vitro models

#### CF and cardiomyocyte isolation.

Cardiomyocytes and CF were isolated as previously described (62). Briefly, adult male Sprague-Dawley rats (250–300 g) were euthanized with either Ketamine (70 mg/kg) and Dexmedetomidine (0.5 mg/kg) or exsanguination under isoflurane. Hearts were immediately excised, trimmed of extraaortic tissue, and retrogradely perfused for 2 minutes in Krebs-Henseleit (KH) Buffer at 37°C and then switched to enzyme buffer 1 (KH buffer containing 0.3 mg/mL collagenase II, 0.3 mg/mL hyaluronidase, and 50 μM CaCl_2_) for 23–25 minutes. After perfusion, the ventricular tissue was excised, minced with scissors, and further digested in enzyme buffer 2 (enzyme buffer 1 supplemented with trypsin IX (MilliporeSigma) 0.6 mg/mL, deoxyribonuclease (Worthington Biochemical) 0.6 mg/mL, and increased CaCl_2_ (MilliporeSigma) to 500 μM) at 37°C for 18 minutes in a shaking water bath. The digestion was stopped with the addition of 10 mL of DMEM (Thermo Fisher Scientific) supplemented with 10% FBS (Gemini Bio-Products), penicillin, and streptomycin (complete media; Thermo Fisher Scientific), filtered through a 200 μm nylon mesh (ELKO Filtering Co.), and centrifuged at 48*g* at room temperature for 5 minutes. The pellet was collected for cardiomyocytes, and rod-shaped cardiomyocytes were purified by going through 0.6% BSA solution. Purified cardiomyocytes were then washed and plated on laminin-coated (Corning) dishes for subsequent applications. The supernatant containing CF was centrifuged at 760*g* at room temperature for 5 minutes. The resulting pellet was resuspended in complete media and plated into 4 10 cm dishes. The media was changed after 2 hours to remove cellular debris and unbound cells. Cells were cultured to confluency within 2–3 days, before being passaged for in vitro experiments. Only Passage 1 (P1) cells were used in this study.

#### HEK293T cell culture and transfection.

HEK293T cells (provided by Keigi Fujiwara at University Texas MD Anderson, Houston, Texas, USA) were maintained in DMEM (GE Healthcare) supplemented with 4.5 g/L glucose, 1 mM sodium pyruvate, 1% L-glutamine, 10% FBS (Thermo Fisher Scientific), 100 U/mL penicillin, 100 μg/mL streptomycin (Corning and Genesee Scientific) at 37°C with 5% CO_2_ in a humidified incubator. For transfection, HEK293T cells were dissociated using Accutase (Innovative Cell Technologies) and plated on 100 mm dishes in 75%–80% confluence condition 1 day before transfection.

Stable HEK293T cells carrying EGFR-GFP and FGFR-Flag were generated by transfecting with EGFR-GFP in PEGFP-N1 and FGFR1 in pWZL Neo Myr Flag, respectively, using Fugene HD transfection reagent (Promega), and selecting with 1,600 μg/mL G-418 (Mediatech/Corning) as done previously (63, 64). Cells stably expressing GFP or Flag were used for control. One month after starting selection under 1600 μg/mL G-418, cell colonies were isolated under the microscope and were separately maintained. Expression levels of EGFR-GFP, GFP, FGFR-Flag, and Flag were confirmed by Western blotting. For EGFR/FGFR transactivation assays, stable cell lines were transiently transfected with α7 nAChR and Myc-tagged NACHO using Xfect Transfection Reagent (Takara Bio) per manufacturer’s protocol. Twenty-four hours after the transfection, cells were detached from the dish using Accutase and replated into the 150 mm dishes for experiments after an additional 24 hours.

#### EGFR transactivation assays.

Seventy-two–hour posttransfected HEK293T cells were pretreated with either α-BTX (Abcam) or BAPTA-AM (Invitrogen and Cayman Chemical) for 10 minutes before activation of nAChR by nicotine. Cells were then collected for immunoblotting. To stimulate FGFR1 in HEK29T cells, cells were treated with 1 ng/mL recombinant human FGF in the presence of 90 μg/mL heparin for 10 minutes before cells were collected for immunoblotting.

#### Cardiac cell coculture experiments.

Cardiomyocytes were isolated and cultured as described above. After 2 days, media from RV cardiac myocytes was collected and used to treat quiesced adult rat cardiac fibroblasts (ARCFs; balanced with M199 media; MilliporeSigma) that were pretreated with α-BTX or vehicle. After 24 hours, cells were trypsinized and counted via hemocytometer. In separate experiments, BrdU incorporation and collagen content were also measured.

#### Knockdown of EGFR siRNA experiments in CFs.

ARCFs isolated from rats were seeded at 50%–70% confluency. Cells were transfected with either EGFR siRNA (sc-108050) or Scrambled siRNA (sc-37007) using Lipofectamine RNAiMAX Transfection Reagent (Thermo Fisher Scientific) according to manufacturer’s protocol. After 24 hours, media was replaced with complete media, followed by serum-free media for another 24 hours, before cells were treated with vehicle, ACh, or nicotine. After 24-hour treatment, cells were trypsinized and collected for protein assay, collagen content measurement, and cell counting.

### In vivo functional assessments

#### Echocardiographic measurement.

Animals were anesthetized with continuous isoflurane inhalation (1.5%–2%), and transthoracic echocardiography was performed as previously described (65). Rodent transducers MS200 and MS250 were used on rats and mice, respectively (Vevo 2100, VisualSonics). Two-dimensional, Doppler, and M-mode recordings were obtained to measure LV fractional shortening and LV and RV dimensions. The pulsed-wave Doppler recording at the right ventricular overflow tract was used to measure pulmonary acceleration time (PAT). Tricuspid annular plane systolic excursion (TAPSE) was measured by use of M-mode across the tricuspid valve annulus at the RV free wall. TAPSE was determined by measuring the excursion of the tricuspid annulus from its highest position to the peak descent during ventricular systole. Tissue Doppler was used to measure the early diastolic velocity of the septum (at mitral annulus) and RV lateral wall (at tricuspid annulus).

#### Invasive hemodynamic measurements.

RVSP, LV systolic pressure (LVSP), and PAP were measured under isoflurane anesthesia (1.5%–2%) using an open-chest technique. Access to the heart was achieved with a laparotomy, followed by dissection of the diaphragm, exposing the thoracic cavity. High-fidelity Millar pressure-volume 1.0F catheter for mice (PVR-1030) or 2.0F catheter for rats (SPR-869) was inserted through a puncture into the apex of the LV and subsequently into the apex of the RV. Steady state recording were acquired for at least 30 seconds, followed by at least 3 successful attempts for vena cava occlusion (2 kHz sampling rate; LabChart 8, ADInstruments). The catheter was then guided into the PA just beyond the pulmonic valve to record the PAP in select animals. Analyses of PV loops were performed off-line. Stoke volume (in relative volume unit [RVU]) derived from the conductance catheter was calibrated by the echocardiogram stroke volume (in mL or μL). RVSP and RVEDP were automatically determined from the steady state measurement, as well as arterial elastance (Ea). From vena cava occlusion, end-systolic elastance (Ees; RV contractility) and end-diastolic elastance (Eed; RV stiffness) were determined. The RV arterial coupling was calculated by the Ees/Ea ratio (66).

### Histology and microscopy

#### Pulmonary vascular remodeling.

PA muscularization was determined by the analysis of H&E-stained sections of rat lung tissue as previously described (67). Slides were scanned into Aperio ScanScope CS (×20 magnification; Leica Biosystems Inc.). The inner and outer lumen diameters of vessels between 30 and 150 μM were measured in a blinded fashion, and percent vessel thickness (reflective of muscularization) was determined with the following equation:

Thirty vessels were averaged per animal, and each animal was considered as *n* = 1. Vessels were also scored for occlusive lesions as open, partially occluded, and fully occluded by a blinded technician. Analysis was represented as percentage of each score divided by total amount of vessels counted.

#### Picrosirius red staining.

Collagen content was visualized by Picrosirius red staining of the RV free wall. Slides were scanned using Aperio ScanScope CS (×20 magnification).

#### Immunofluorescence staining and quantification.

Cryosections of the rodent RV free wall (5 μm) were fixed with 4% paraformaldehyde, permeabilized with Triton-X (0.1%), blocked with 10% goat serum in PBS, and incubated with primary antibodies against pEGFR (1:50; Phospho-EGFR, Tyr1068, 1H12, Cell Signalling Technologies) and vimentin (1:100; E-5, Santa Cruz Biotechnology, sc-373717) overnight, followed by appropriate secondary antibodies for 1 hour (Jackson ImmunoResearch). Sections were also stained with wheat germ agglutinin (WGA, 1:50, W32566, Invitrogen) and Hoechst (33342, 1:1000 in PBS; Thermo Fisher Scientific) counterstaining. Image acquisition was performed on Zeiss LSM780 confocal microscope system. Quantification was performed using ImageJ software (NIH). The area positive for pEGFR was divided by total area, measured over 3 randomly areas in the RV. FFPE human RV sections were deparaffinized and rehydrated as follows: 100% xylene for 10 minutes twice, 100% ethanol for 10 minutes twice, 95% ethanol for 5 minutes twice, 70% ethanol for 5 minutes once, 50% ethanol for 5 minutes once, and water for 5 minutes twice. The slides were then transferred into 200 mL of prewarmed (94°C–96°C) 1× target retrieval solution (S1699, Agilent Dako) and steamed for 30 minutes; they were then allowed to cool at room temperature. The sections were then washed in 1× TBS for 10 minutes, blocked with a mix of 10% goat serum and 5% BSA in 1× TBS for 1 hour, and incubated with primary antibodies against vimentin (1:50) and α7 nAChR (1:50; Alomone Labs, ANC-007) overnight. The next day, the sections were rinsed in 1× TBS and incubated with fluorescent secondary antibodies (1:2000) for 2 hours, followed by staining of nuclei with Hoechst (1:2000). They were mounted with ProLong Gold Antifade (Thermo Fisher Scientific). Image acquisition was performed on Zeiss LSM780 confocal microscope.

#### Adult rat CF immunofluorescence staining and quantification.

Cells were plated at ~70% confluence on glass coverslips for 24 hours. Cells were fixed with 4% paraformaldehyde, permeabilized with 0.1% Triton-X (for pEGFR staining), and blocked in 5% goat serum for 30 minutes. After blocking, samples were incubated with anti-pEGFR antibody (1:100) or α7 nAChR (1:50), washed, and then incubated for 1 hour with appropriate fluorescent secondary antibody (1:200–1:300, Jackson ImmunoResearch), nuclear counterstained with Hoechst or DAPI, and mounted with Prolong Gold Antifade. Images were obtained with either a confocal Zeiss LMS780 laser-scanning microscope (×20 or ×40 magnification) of Nikon Eclipse E400 IF microscope. Confocal images were analyzed using Zen Blue (Zeiss) and ImageJ software (NIH). For each cell surface marker, slides were imaged at identical wide-field (magnification, scan speed, laser power, detector gain, and pinhole diameter) settings. The intensity of pEGFR was measured outlining the cells. Some images were then analyzed on NS Elements (Nikon) by measuring intensity of FITC channel divided over the measured area. Three to 5 images per animals were used and averaged together as 1.

#### In vitro live cell α7 nAChR staining via α-BTX.

Adult rat CF and cardiomyocytes were plated onto glass chamber slides overnight and then incubated with α-BTX-FITC (1:2000, B13422, Thermo Fisher Scientific) for 2 hours in a 37°C humidified chamber. Cells were then fixed with 4% PFA, counterstained with Hoechst, and mounted and imaged on a confocal Zeiss LMS780 laser-scanning microscope.

#### Calcium imaging.

Changes in [Ca^2+^]_c_ in response to nicotine stimulation were observed in live ARCFs using a Nikon TE2000 live cell epifluorescence microscope (Nikon) equipped with Retiga EXi camera (QImaging). Cells were plated on glass-bottom 35 mm dishes (Matek and Matsunami Glass USA Inc.), loaded with a Ca^2+^-sensitive dye Fluo-3 (Biotium), and used for observation. Cell culture medium was replaced by Tyrode solution (mm): NaCl, 136.9; KCl, 5.4; CaCl_2_, 2; MgCl_2_, 0.5; NaH_2_PO_4_, 0.33; HEPES, 5; glucose, 5 (pH 7.40), adjusted with NaOH.

### Cell proliferation assays

Cells were trypsinized and counted as previously described (17). Cells seeded with equal density were allowed to adhere overnight and were then serum-starved with serum-free DMEM supplemented 10 μg/mL insulin, 5.5 μg/mL transferrin, 5 ng/mL sodium selenite (ITS, Corning), and penicillin and streptomycin. After 24 hours, cells were pretreated with inhibitors for 30 minutes and then treated with agonists for another 24 hours. Cells were trypsinized and counted with a hemocytometer. For BrdU incorporation experiments, cells were seeded equally in a 96-well plate, serum starved for 24 hours, and then treated with inhibitors/agonists and with BrdU label, concomitantly. After 24 hours, cells were fixed and assayed for BrdU content per manufacturer’s directions (Sigma-Aldrich).

### Biochemical and molecular biology assays

#### Collagen content measurement.

Collagen content was measured using Sircol collagen dye binding assay according to manufacturer’s instructions (Accurate). Equal amounts of protein or homogenates were mixed with equal volume of Sircol dye reagent for 30 minutes with agitation. The collagen-dye complex was centrifuged at 14,500*g* at 4°C for 10 minutes, and unbound dye was aspirated. The remaining pellet was washed with ice cold acid-salt wash reagent (acetic acid, sodium chloride, and surfactants). Samples were then centrifuged again at 14,500*g* at 4°C for 10 minutes. The wash reagent was aspirated, and the remaining pellet was dissolved by adding the alkali reagent (0.5M sodium hydroxide). After 5 minutes of incubation, collagen content was measured for absorbance at 555 nm.

#### ELISA assays.

Levels of ACh (mouse, NBP2-66389, Novus Biologicals; rat, LS-F27870, LifeSpan BioSciences) and AChE activity (KA4132, Novus Biologicals) were measured in homogenized tissue per manufacturer’s directions.

#### Quantitative PCR.

Total RNA was extracted from tissue with RNeasy Mini Kit (Qiagen). The cDNA was made by High-Capacity cDNA Reverse Transcription Kit (Thermo Fisher Scientific) and used for quantitative PCR (qPCR) reactions. qPCR was performed on Step One Plus Real-time PCR System (Applied Biosystems) with the SsoAdvanced Universal SYBR Green Supermix (Bio-Rad). Data for each gene were normalized to GAPDH (rat) or actin (human and mouse). Real-time PCR was performed using the primer sets listed in Supplemental Table 7.

#### Western blotting.

Animal tissues were homogenized at 4°C in homogenization buffer (20 mM HEPES, 250 mM sucrose, 100 mM NaCl, 0.2 mM EDTA, and 0.2 mM EGTA and supplemented with protease and phosphatase inhibitors) as previously described (68, 69). Homogenates were centrifuged at 2500*g* at 4°C for 10 minutes. The pellet was discarded, and supernatant was then centrifuged at 21,000*g* for 10 minutes at 4°C. The resulting supernatant was collected for protein analysis. Total protein concentration was determined with DC Protein Assay (Bio-Rad). HEK293T cells were lysed with 1× lysis buffer (Cell Signaling Technology) containing protease inhibitor cocktail (Sigma-Aldrich) and 1 mM phenylmethylsulfonyl fluoride (PMSF). Protein concentrations were determined by Pierce BCA Protein Assay Kit (Thermo Fisher Scientific).

Equal amounts of total proteins were resolved on separating gels by SDS-PAGE, followed by transfer to polyvinylidene difluoride or nitrocellulose membrane, and immunoblots were visualized with Bio-Rad Imager Chemidoc or with Odyssey infrared imaging system (LI-COR Biotechnology). Quantitative densitometry was performed using ImageJ (NIH, available online at http://rsb.info.nih.gov/ij/).

### Statistics

Statistical analyses were performed using GraphPad Prism 9.1.0.221 (La Jolla, www.graphpad.com). Data are presented as mean ± SEM; *P* < 0.05 was considered statistically significant. Normality of data was checked, and either log-transformation or nonparametric testing was performed if data were not normally distributed. Mann-Whitney *U* test, unpaired 2-tailed Student’s *t* test, or 1- or 2-way ANOVA with Bonferroni post hoc test were used when appropriate. Figure legends specify the tests used for each experiment.

### Study approval

All animal protocols were approved by the IACUCs at the Providence VA Medical Center, Rhode Island Hospital, and University of Minnesota. Collection and sharing of human tissue were approved by the Cleveland Clinic IRB and adhered to all required ethical standards for human subject research. Use of human tissue was approved by the Providence VA Medical Center IRB.

## Author contributions

GC, JO, and AV designed research studies; AV, DDSGB, AFN, PZ, TJM, IP, MWC, BSJ, JO, and GC conducted experiments and acquiring data; AV, DDSGB, AFN, PZ, ARM, JO, RTC, UM, and GC analyzed or interpreted data; EH provided reagents; and AV, DDSGB, AFN, PZ, ARM, RTC, BSJ, EH, UM, BSJ, JO, and GC contributed to writing and editing the manuscript. The method used in assigning the authorship order among co–first authors was based on relative contributions in experimental design; conducting experiments, analyses, and interpretation of data; and writing of the manuscript.

## Supplementary Material

Supplemental data

## Version 1 05/11/2021

In-Press Preview

## Version 2 06/22/2021

Electronic publication

## References

[1] Frangogiannis NG (2019). Cardiac fibrosis: Cell biological mechanisms, molecular pathways and therapeutic opportunities. Mol Aspects Med.

[2] Frangogiannis NG (2017). Fibroblasts and the extracellular matrix in right ventricular disease. Cardiovasc Res.

[3] Tallquist MD, Molkentin JD (2017). Redefining the identity of cardiac fibroblasts. Nat Rev Cardiol.

[4] Travers JG (2016). Cardiac fibrosis: the fibroblast awakens. Circ Res.

[5] Andersen S (2019). Right ventricular fibrosis. Circulation.

[6] Bogaard HJ, Voelkel NF (2019). Is myocardial fibrosis impairing right heart function?. Am J Respir Crit Care Med.

[7] Egemnazarov B (2018). Right ventricular fibrosis and dysfunction: Actual concepts and common misconceptions. Matrix Biol.

[8] Clements RT (2019). Treatment of pulmonary hypertension with angiotensin II receptor blocker and neprilysin inhibitor sacubitril/valsartan. Circ Heart Fail.

[9] Taraseviciene-Stewart L (2006). Simvastatin causes endothelial cell apoptosis and attenuates severe pulmonary hypertension. Am J Physiol Lung Cell Mol Physiol.

[10] Simonneau G (2019). Haemodynamic definitions and updated clinical classification of pulmonary hypertension. Eur Respir J.

[11] Bogaard HJ (2009). Chronic pulmonary artery pressure elevation is insufficient to explain right heart failure. Circulation.

[12] Kusakari Y (2017). Impairment of excitation-contraction coupling in right ventricular hypertrophied muscle with fibrosis induced by pulmonary artery banding. PLoS One.

[13] Rain S (2016). Right ventricular myocardial stiffness in experimental pulmonary arterial hypertension: relative contribution of fibrosis and myofibril stiffness. Circ Heart Fail.

[14] Gomez-Arroyo J (2014). Differences in right ventricular remodeling secondary to pressure overload in patients with pulmonary hypertension. Am J Respir Crit Care Med.

[15] Mehta BB (2015). Detection of elevated right ventricular extracellular volume in pulmonary hypertension using accelerated and navigator-gated look-locker imaging for cardiac T1 estimation (ANGIE) cardiovascular magnetic resonance. J Cardiovasc Magn Reson.

[16] Simpson CE, Hassoun PM (2019). Myocardial fibrosis as a potential maladaptive feature of right ventricle remodeling in pulmonary hypertension. Am J Respir Crit Care Med.

[17] Vang A (2017). Effect of alpha7 nicotinic acetylcholine receptor activation on cardiac fibroblasts: a mechanism underlying RV fibrosis associated with cigarette smoke exposure. Am J Physiol Lung Cell Mol Physiol.

[18] Albuquerque EX (2009). Mammalian nicotinic acetylcholine receptors: from structure to function. Physiol Rev.

[19] Zheng Y (2007). Nicotine stimulates human lung cancer cell growth by inducing fibronectin expression. Am J Respir Cell Mol Biol.

[20] Improgo MR (2011). Nicotinic acetylcholine receptor-mediated mechanisms in lung cancer. Biochem Pharmacol.

[21] Cucina A (2012). Nicotine stimulates proliferation and inhibits apoptosis in colon cancer cell lines through activation of survival pathways. J Surg Res.

[22] Li JM (2004). Nicotine enhances angiotensin II-induced mitogenic response in vascular smooth muscle cells and fibroblasts. Arterioscler Thromb Vasc Biol.

[23] Cucina A (2008). Nicotine inhibits apoptosis and stimulates proliferation in aortic smooth muscle cells through a functional nicotinic acetylcholine receptor. J Surg Res.

[24] Heeschen C (2002). A novel angiogenic pathway mediated by non-neuronal nicotinic acetylcholine receptors. J Clin Invest.

[25] Tsuneki H (2004). Nicotinic enhancement of proliferation in bovine and porcine cerebral microvascular endothelial cells. Biol Pharm Bull.

[26] Toba M (2014). Temporal hemodynamic and histological progression in Sugen5416/hypoxia/normoxia-exposed pulmonary arterial hypertensive rats. Am J Physiol Heart Circ Physiol.

[27] Jaldety Y (2012). Sperm epidermal growth factor receptor (EGFR) mediates α7 acetylcholine receptor (AChR) activation to promote fertilization. J Biol Chem.

[28] Grisanti LA (2017). Cardiac GPCR-mediated EGFR transactivation: impact and therapeutic implications. J Cardiovasc Pharmacol.

[29] Gu S (2016). Brain α7 nicotinic acetylcholine receptor assembly requires NACHO. Neuron.

[30] Uteshev VV (2012). α7 nicotinic ACh receptors as a ligand-gated source of Ca(2+) ions: the search for a Ca(2+) optimum. Adv Exp Med Biol.

[31] Orr-Urtreger A (1997). Mice deficient in the alpha7 neuronal nicotinic acetylcholine receptor lack alpha-bungarotoxin binding sites and hippocampal fast nicotinic currents. J Neurosci.

[32] Kanazawa H (2010). Heart failure causes cholinergic transdifferentiation of cardiac sympathetic nerves via gp130-signaling cytokines in rodents. J Clin Invest.

[33] Roy A (2013). Cardiomyocyte-secreted acetylcholine is required for maintenance of homeostasis in the heart. FASEB J.

[34] Rocha-Resende C (2012). Non-neuronal cholinergic machinery present in cardiomyocytes offsets hypertrophic signals. J Mol Cell Cardiol.

[35] da Silva Goncalves Bos D (2018). Contribution of impaired parasympathetic activity to right ventricular dysfunction and pulmonary vascular remodeling in pulmonary arterial hypertension. Circulation.

[36] Colombo SF (2013). Biogenesis, trafficking and up-regulation of nicotinic ACh receptors. Biochem Pharmacol.

[37] Jaffre F (2009). Serotonin and angiotensin receptors in cardiac fibroblasts coregulate adrenergic-dependent cardiac hypertrophy. Circ Res.

[38] Kleine-Brueggeney M (2014). Alpha1a-adrenoceptor genetic variant induces cardiomyoblast-to-fibroblast-like cell transition via distinct signaling pathways. Cell Signal.

[39] Eguchi S (1998). Calcium-dependent epidermal growth factor receptor transactivation mediates the angiotensin II-induced mitogen-activated protein kinase activation in vascular smooth muscle cells. J Biol Chem.

[40] Murasawa S (1998). Angiotensin II type 1 receptor-induced extracellular signal-regulated protein kinase activation is mediated by Ca2+/calmodulin-dependent transactivation of epidermal growth factor receptor. Circ Res.

[41] Sanchez-Gonzalez P (2010). Calmodulin-mediated regulation of the epidermal growth factor receptor. FEBS J.

[42] Heeschen C (2001). Nicotine stimulates angiogenesis and promotes tumor growth and atherosclerosis. Nat Med.

[43] Oakes JM (2020). Effects of chronic nicotine inhalation on systemic and pulmonary blood pressure and right ventricular remodeling in mice. Hypertension.

[44] Maron BA (2016). Association of borderline pulmonary hypertension with mortality and hospitalization in a large patient cohort: insights from the Veterans Affairs Clinical Assessment, Reporting, and Tracking Program. Circulation.

[45] Maron BA (2013). Clinical profile and underdiagnosis of pulmonary hypertension in US veteran patients. Circ Heart Fail.

[46] Assad TR (2017). Prognostic effect and longitudinal hemodynamic assessment of borderline pulmonary hypertension. JAMA Cardiol.

[47] Strange G (2019). Threshold of pulmonary hypertension associated with increased mortality. J Am Coll Cardiol.

[48] Huston JH (2019). Association of mild echocardiographic pulmonary hypertension with mortality and right ventricular function. JAMA Cardiol.

[49] van de Veerdonk MC (2011). Progressive right ventricular dysfunction in patients with pulmonary arterial hypertension responding to therapy. J Am Coll Cardiol.

[50] Trip P (2015). Clinical relevance of right ventricular diastolic stiffness in pulmonary hypertension. Eur Respir J.

[51] Thenappan T (2018). Pulmonary arterial hypertension: pathogenesis and clinical management. BMJ.

[52] Bogaard HJ (2010). Adrenergic receptor blockade reverses right heart remodeling and dysfunction in pulmonary hypertensive rats. Am J Respir Crit Care Med.

[53] Gomez-Arroyo J (2015). Iloprost reverses established fibrosis in experimental right ventricular failure. Eur Respir J.

[54] Kojonazarov B (2017). p38 MAPK inhibition improves heart function in pressure-loaded right ventricular hypertrophy. Am J Respir Cell Mol Biol.

[55] Budas GR (2018). ASK1 inhibition halts disease progression in preclinical models of pulmonary arterial hypertension. Am J Respir Crit Care Med.

[56] Crnkovic S (2019). Disconnect between fibrotic response and right ventricular dysfunction. Am J Respir Crit Care Med.

[57] Hsu S (2018). Right ventricular myofilament functional differences in humans with systemic sclerosis-associated versus idiopathic pulmonary arterial hypertension. Circulation.

[58] Wang Y (2014). The duplicated α7 subunits assemble and form functional nicotinic receptors with the full-length α7. J Biol Chem.

[59] Carter RE, Sorkin A (1998). Endocytosis of functional epidermal growth factor receptor-green fluorescent protein chimera. J Biol Chem.

[60] Boehm JS (2007). Integrative genomic approaches identify IKBKE as a breast cancer oncogene. Cell.

[61] Egemnazarov B (2015). Pressure overload creates right ventricular diastolic dysfunction in a mouse model: assessment by echocardiography. J Am Soc Echocardiogr.

[62] Zhang P (2011). Regulator of G protein signaling 2 is a functionally important negative regulator of angiotensin II-induced cardiac fibroblast responses. Am J Physiol Heart Circ Physiol.

[63] O-Uchi J (2013). Overexpression of ryanodine receptor type 1 enhances mitochondrial fragmentation and Ca2+-induced ATP production in cardiac H9c2 myoblasts. Am J Physiol Heart Circ Physiol.

[64] O-Uchi J (2014). Adrenergic signaling regulates mitochondrial Ca2+ uptake through Pyk2-dependent tyrosine phosphorylation of the mitochondrial Ca2+ uniporter. Antioxid Redox Signal.

[65] McCullough DJ (2014). NS1619-induced vasodilation is enhanced and differentially mediated in chronically hypoxic lungs. Lung.

[66] da Silva Gonçalves Bos D (2017). Renal denervation reduces pulmonary vascular remodeling and right ventricular diastolic stiffness in experimental pulmonary hypertension. JACC: Basic to Translational Science.

[67] Casserly B (2011). C-type natriuretic peptide does not attenuate the development of pulmonary hypertension caused by hypoxia and VEGF receptor blockade. Life Sci.

[68] Choudhary G (2011). Bosentan attenuates right ventricular hypertrophy and fibrosis in normobaric hypoxia model of pulmonary hypertension. J Heart Lung Transplant.

[69] Vang A (2010). Activation of endothelial BKCa channels causes pulmonary vasodilation. Vascul Pharmacol.

