# Supplementary material for: α7 Nicotinic acetylcholine receptor mediates right ventricular fibrosis and diastolic dysfunction in pulmonary hypertension
Source: JCI Insight. 2021 Jun 22;6(12):e142945. doi: 10.1172/jci.insight.142945 (PMC8262476; doi:10.1172/jci.insight.142945)
Supplement: Supplemental data [file jciinsight-6-142945-s216.pdf]

Table S1: Echocardiography parameters in the rat Sugen/Hypoxia pulmonary hypertension model at 3 and 7 wks.

	Control 3 wks (n=9)	PH 3 wks (n=11)	P-value	Control 7 wks (n=6)	PH 7 wks (n=5)	P-value
Cardiac output (mL/min)	84±6.9	58±4.9	0.0053	77±6.5	51±11.9	0.07
Heart rate (bpm)	357±7.9	373±10.9	0.27	350±17.3	309±4.5	0.06
TAPSE (mm)	2.2±0.10	1.5±0.12	0.0008	2.9±0.27	2.1±0.12	0.02
RV s'(mm/s)	46.3±2.9	35.7±2.3	0.01	56.8±5.1	42±3.3	0.04
RV e' (mm/s)	-33.8±2.9	-22.6±2.3	0.008	-41.3±2.9	-16.2±2.3	0.0001
RV E/A	0.50±0.04	0.74±0.17	0.16	0.54±0.08	0.44±0.05	0.36
LV EF (%)	80±1.8	83±3.7	0.51	75±2.3	85±3.3	0.03
LV E/A	1.3±0.15	1.1±0.07	0.15	1.3±0.11	1.1±0.14	0.28
PAT (ms)	34.7±2.4	16.7±0.9	<0.0001	28.8±1.4	17.4±1.5	0.0004
RV wall thickness (mm)	0.5±0.06	1.4±0.09	<0.0001	0.6±0.03	1.7±0.08	<0.0001

All data presented as mean ± SEM. Unpaired t-test or Mann-Whitney test; Abbreviations: TAPSE= tricuspid annular plane systolic excursion; RV s'= doppler tissue imagining tricuspid lateral annular systolic velocity wave (s'); RV e'= lateral tricuspid annulus velocity during early filling (e'); EF= ejection fraction; E/A= early and late filling waves, indication of RV diastolic function; PAT = pulmonary acceleration time.

Table S2: Characteristics of human subjects:

Gender	Disease	Age	Race
**Controls**			
Female	Control	81	
Female	Control	50	Caucasian
Male	Control	49	Black
Female	Control	76	Caucasian
**PAH**			
Male	IPAH	61	
Female	IPAH	47	
Male	IPAH	27	Caucasian
Female	IPAH	17	
Male	IPAH	50	Caucasian
Female	PAH	56	

Table S3: Echocardiography parameters before and after mecamylamine treatment (starting at week 3).

	PH-Veh Pre (n=7)	PH-Veh Post (n=7)	P-value	PH-Mec Pre (n=9)	PH-Mec Post (n=9)	P-value
Cardiac output (mL/min)	68.3±5.8	67.7±3.9	0.88	60.5±3.8	71.4±5.9	0.12
RV Stroke volume (mL)	0.18±0.01	0.19±0.01	0.19	0.16±0.01	0.20±0.01	0.02
Heart rate (bpm)	388±5.0	358±8.5	0.02	379±6.5	351±6.0	0.0006
TAPSE (mm)	1.9±0.13	1.7±0.06	0.17	1.8±0.11	1.8±0.14	0.89
RV s'(mm/s)	31.1±2.5	30.4±3.1	0.82	34.1±2.6	35.5±2.7	0.79
RV e' (mm/s)	-17.6±5.4	-19.5±3.2	0.96	-13.3±1.2	-21.0±2.3	0.04
RV E/e'	-45.2±17.7	-15.9±2.7	0.25	-30.3±9.2	-18.6±3.6	0.56
RV E/A	0.77±0.32	0.41±0.05	0.38	0.61±0.14	0.54±0.09	0.25
LV EF (%)	79.4±3.1	76.4±2.5	0.35	74.02±3.1	76.1±3.1	0.34
LV E/A	0.71±0.23	0.98±0.06	0.31	0.79±0.20	1.3±0.12	0.03
PAT (ms)	23.3±1.7	21.1±1.4	0.04	19.4±1.6	20.2±1.3	0.73
RV wall thickness (mm)	1.3±0.10	1.4±0.13	0.32	1.3±0.11	1.1±0.11	0.01

All data are presented as mean ± SEM; Paired t-test or Wilcoxon; Abbreviations: MEC= mecamylamine; TAPSE= tricuspid annular plane systolic excursion; RV s'= doppler tissue imagining tricuspid lateral annular systolic velocity wave (s'); RV e'= lateral tricuspid annulus velocity during early filling (e'); E/A= early and late filling waves, indication of RV diastolic function; PAT = pulmonary acceleration time.

Table S4: Echocardiography parameters before and after mecamylamine treatment (starting at week 5).

	PH-Veh Pre (n=6)	PH-Veh Post (n=6)	P-value	PH-Mec Pre (n=10)	PH-Mec Post (n=10)	P-value
Cardiac output (mL/min)	66.3±6.4	64.0±4.1	0.59	56.6±4.1	78.4±5.0	0.0001
Stroke volume (mL)	0.19±0.02	0.18±0.01	0.74	0.16±0.01	0.22±0.01	0.0001
Heart rate (bpm)	354±10.2	347±5.5	0.56	361±8.3	359±6.8	0.83
TAPSE (mm)	2.1±0.17	2.0±0.18	0.78	2.1±0.17	2.2±0.19	0.52
RV s'(mm/s)	35.7±2.4	32.4±2.4	0.55	36.1±1.6	34.8±1.8	0.52
RV e' (mm/s)	-21.5±1.9	-27.0±5.7	0.57	-21.7±2.0	-28.1±3.9	0.08
RV E/e'	-18.7±3.3	-14.2±2.3	0.36	-17.7±3.1	-11.8±2.1	0.04
RV E/A	0.44±0.03	0.41±0.08	0.59	0.49±0.04	0.40±0.03	0.23
LV EF (%)	75.8±2.0	77.5±2.9	0.47	81.5±2.7	80.4±1.2	0.70
LV E/A	1.1±0.05	1.1±0.12	0.89	1.3±0.14	1.1±0.11	0.16
PAT (ms)	15.0±0.9	15.5±0.9	0.72	16.4±1.2	20.0±1.4	0.04
RV wall thickness (mm)	1.4±0.18	1.3±0.09	0.22	1.2±0.11	1.0±0.10	0.01

All data are presented as mean ± SEM; Paired t-test or Wilcoxon; Abbreviations: MEC= mecamylamine; TAPSE= tricuspid annular plane systolic excursion; RV s'= doppler tissue imagining tricuspid lateral annular systolic velocity wave (s'); RV e'= lateral tricuspid annulus velocity during early filling (e'); E/A= early and late filling waves, indication of RV diastolic function; PAT = pulmonary acceleration time.

Table S5: Echocardiography parameters in wild-type and α7nAChR knockout mice after pulmonary artery banding

	WT Sham (n=18)	WT PAB (n=26)	KO Sham (n=9)	KO PAB (n=19)	P-value		
					Interaction	WT Sham vs. PAB	KO Sham vs. PAB
Cardiac output (mL/min)	19.7±0.7	17.8±1.0	13.8±1.1	15.2±1.1	0.14	0.86	0.99
Heart rate (bpm)	501±14.5	476±9.6	481±10.3	460±10.9	0.88	0.66	0.99
TAPSE (mm)	1.0±0.06	0.99±0.06	1.1±0.04	0.95±0.06	0.48	0.99	0.99
RV s'(mm/s)	23.0±1.9	16.1±0.7	22.2±1.2	19.3±1.2	0.14	0.001	0.82
RV e' (mm/s)	-18.1±1.6	-16.6±2.0	-17.7±1.4	-15.6±1.1	0.84	0.99	0.99
RV E/A	0.63±0.05	1.3±0.14	0.62±0.04	0.86±0.07	0.06	0.0008	0.02
LV EF (%)	62±2.7	68±2.1	64±3.4	67±2.8	0.70	0.71	0.99
LV E/A	1.5±0.05	1.3±0.08	1.5±0.1	1.6±0,.07	0.19	0.99	0.99
Peak Velocity (mm/s)	-720±28.9	-1355±128.7	-756±27.4	-1493±164	0.67	0.0007	0.0009
RV wall thickness (mm)	0.28±0.02	0.37±0.03	0.31±0.02	0.43±0.06	0.58	0.38	0.14

All data are presented as mean ± SEM; Two-way ANOVA for repeated measurements followed by Bonferroni post-hoc. Abbreviations: WT = wild type; KO = knockout; PAB= pulmonary artery banded; TAPSE = tricuspid annular plane systolic excursion; RV s' = doppler tissue imagining tricuspid lateral annular systolic velocity wave (s'); RV e' = lateral tricuspid annulus velocity during early filling (e'); E/A = early and late filling waves, indication of RV diastolic function; EF = ejection fraction.

Table S6: List of Antibodies

Antibody	Application(s)	Company (Catalog#)
EGFR (C74B9)	WB, ICC	Cell Signaling Technology (#2646)
Phospho-EGFR (Tyr1068) (1H12)	WB, ICC	Cell Signaling Technology (#2236)
GFP (D5.1)	WB	Cell Signaling Technology (#2956)
FGF Receptor 1 (D8E4)	WB	Cell Signaling Technology (#9740)
Phospho-FGF Receptor (Tyr653/654) (55H2)	WB	Cell Signaling Technology (#3476)
α7 nAChR	WB, ICC, IHC	Alomone Labs (ANC-007)
Tubulin (clone B-5-1-2)	WB	Sigma-Aldrich (T5168)
Vimentin (V9)	WB, ICC, IHC	Sigma-Aldrich (V6630)
Myc (Clone 9E10)	WB	BioLegend (626802)
Vimentin (E-5)	WB, ICC, IHC	Santa Cruz Biotechnology (sc-373717)
GAPDH (14C10)	WB	Cell Signaling Technology (#2118)

WB: Western Blot, ICC: Immunocytochemistry, IHC: Immunohistochemistry

Table S7: Primers

Rat Primers	Oligonucleotide Sequences (5'-3')	Human Primers	Oligonucleotide Sequences (5'-3')
Pro Collagen I	F: GTGTTCAAGGTGGCAAAGGT	Pro Collagen I	F: TCCAAAGGAGAGAGCGGTAA
	R: CTTCTCCAGCGGTACCTGAG		R: CAGATCCAGCTTCCCCATTA
α7 nAChR	F: TCCCTCCAGGCATATTCAAG	α7 nAChR	F: TTCTCTCTCAGAGGGGACCA
	R: AGTTTGCACTGCTGCACATC		R: GAGCGTTAGTGCTGGAAAGG
ChAT	F: CGAGCCTTGTTGACATGAGA	ChAT	F: TTTTGTGAGAGCCGTGACTG
	R: TTCTGTAGGAGGCTGGGCTA		R: CATCCTTCAGGAGCAGAAGC
ChT	F: CAAGACCAAGGAGGAAGCAG	ChT	F: CACCATGTGCATCAGGAAAC
	R: GGACATGACAGCAGCAGAAA		R: TTGCCCAACAGAATGGTACA
VAChT	F: TGGTCATTCTGCAAGAGCAC	VAChT	F: TCTCGAAGCCCAGCTATGTT
	R: TCATGGAGAAGGGATTCCAG.		R: CTGGAAGCCAGAGGACTACG
GAPDH	F: CGCTAACATCAAATGGGGTG	Actin	F: TCCCTGGAAAAGAGCTACGA
	R: TTGCTGACAATCTTGAGGGAG		R: TGCTGTTGTAGGTGGTCTCG

Mouse Primers	Oligonucleotide Sequences (5'-3')
α7 nAChR	F: GCACCTCATGCATGGTACAC
	R: TCCACTCACTGCAGATCACC
Actin	F: GCATGCAGAAGGAGATCACA
	R: ACATCTGCTGGAAGGTGGAC

Figure S1

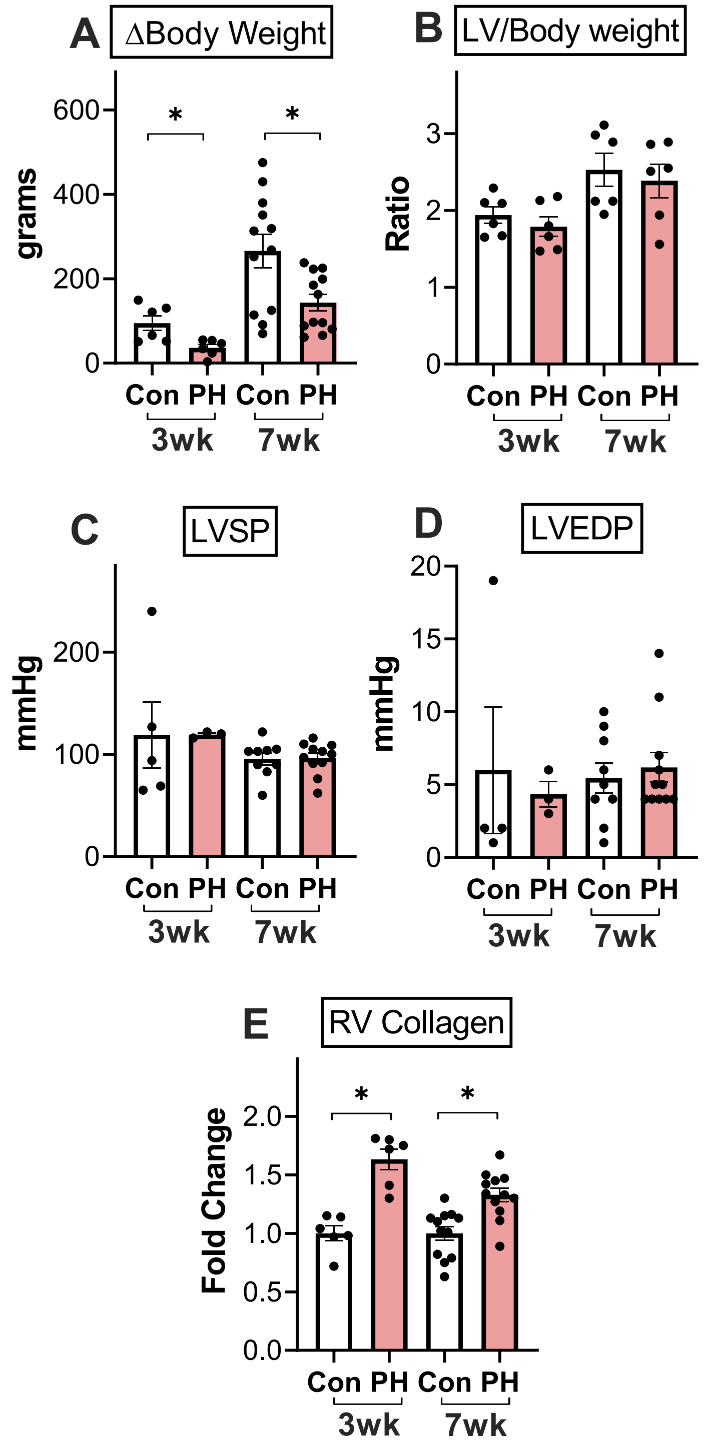

Figure S1. Body weight and cardiac changes in the Sugen/Hypoxia rat PH model. Experimental design as in Figure 1. (**A**) Body weight change between Control and PH groups at 3 (n=6 each) and 7 wks (n=12 each) compared to baseline. (**B**) LV weight divided by body weight (n=6 each). (**C**) Invasively measured LV systolic and (**D**) end diastolic pressures (3 wks n=5/3; and 7 wks n=9/11). (**E**) RV collagen measured by Sircol assay (n=6-12). Mean ± SEM. Two-tail *T*-tests were performed between control and PH at 3 and 7 wks; *$P < 0.05$. LV: left ventricle, LVSP: left ventricular systolic pressure, LVEDP: left ventricular end diastolic pressure.

Figure S2

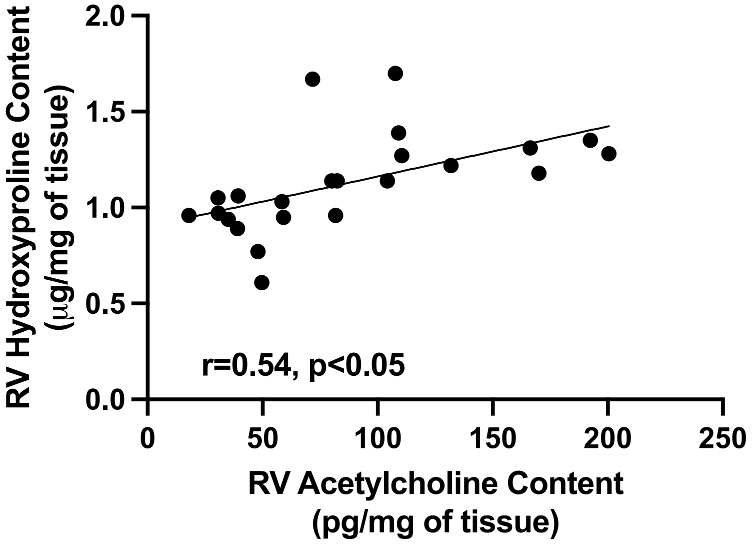

Figure S2. Correlation between ACh and Collagen in the RV.
Correlation between Acetylcholine content and RV collagen as assessed by hydroxyproline content in the cohort of control and PH rats. Both timepoints of 3 weeks and 7 weeks included.

Figure S3

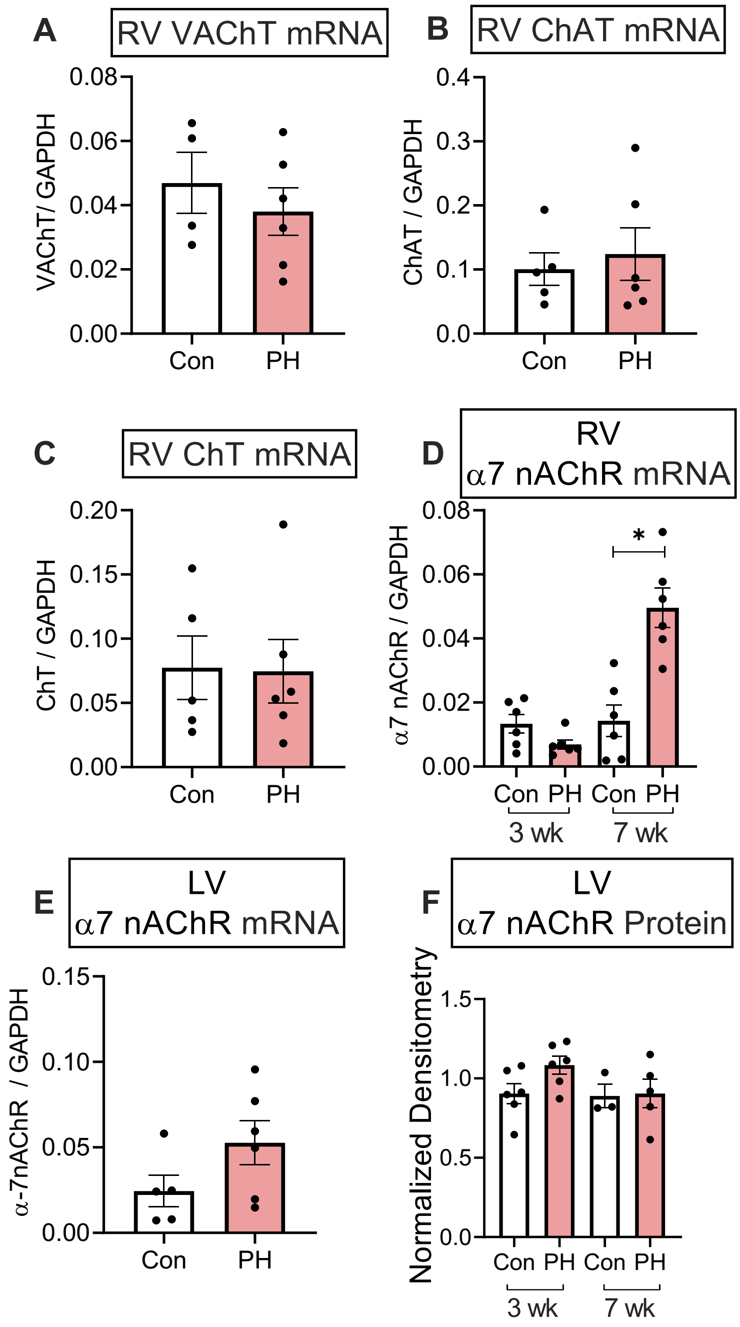

Figure S3. mRNA and protein expression of genes related to ACh synthesis andd α7 nAChR in the RV and LV of the rat PH model

Experimental design as in Figure 1A. mRNA expression of **(A)** vesicular acetylcholine transporter (n=4/6), **(B)** choline acetyltransferase (n=5/6) and (**C**) choline transporter (n=5/6) and in the RV from control and PH rats at 7 wks. α7 nAChR mRNA expression in the **(D)** RV and **(E)** LV of control and PH rats. **(F)** α7 nAChR protein expression in the LV of control and PH rats. Mean ± SEM. Mann-Whitney U test was performed between control and PH; * $p<0.05$. PH: pulmonary hypertension, VAChT: vesicular acetylcholine transporter, ChAT: choline acetyltransferase, ChT: choline transporter.

Figure S4

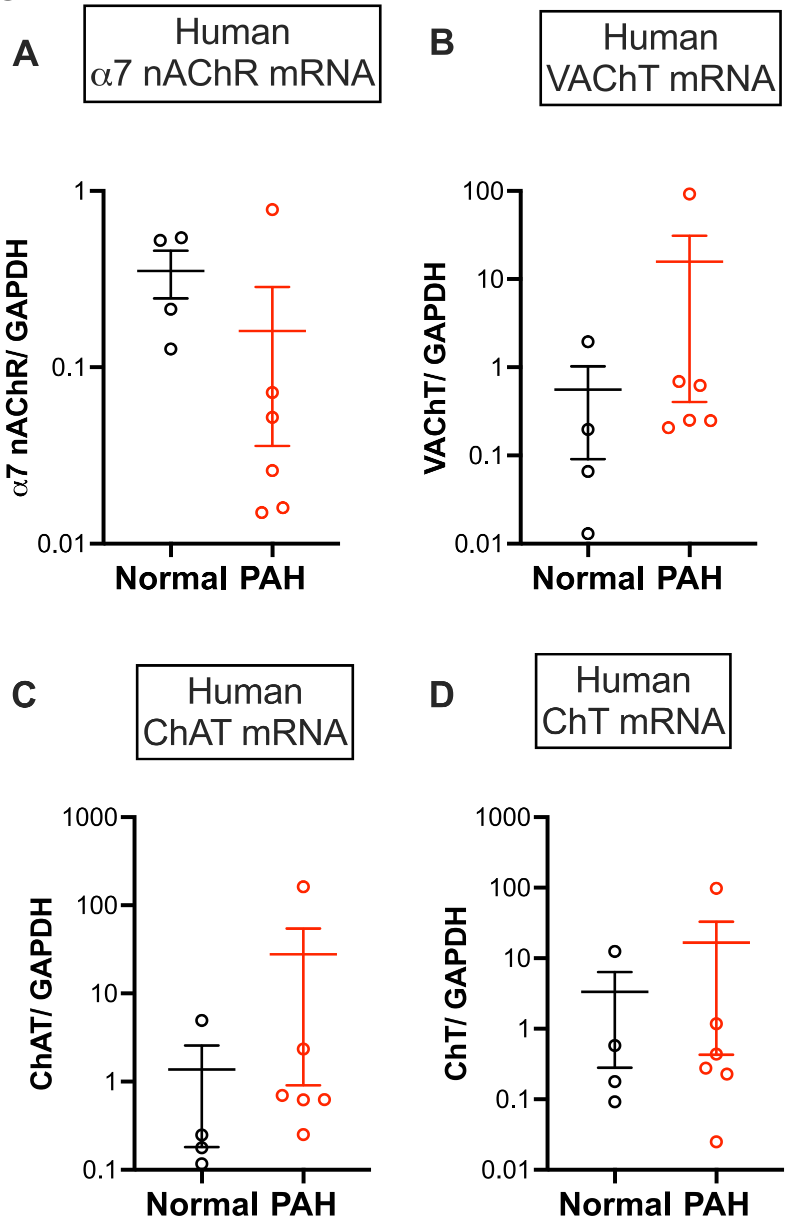

Figure S4. mRNA expression of α7nAChR, ACh synthesis and production related genes in human PAH

mRNA expression of (**A**) α7nAChR (**B**) vesicular acetylcholine transporter, (**B**) choline acetyltransferase expression and (**C**) choline transporter in the RV from control and PAH patients. Mean ± SEM. N=4/5. Mann Whitney test was performed between control and PAH. PAH: pulmonary arterial hypertension, VAChT: vesicular acetylcholine transporter, ChAT: choline acetyltransferase, ChT: choline transporter.

Figure S5

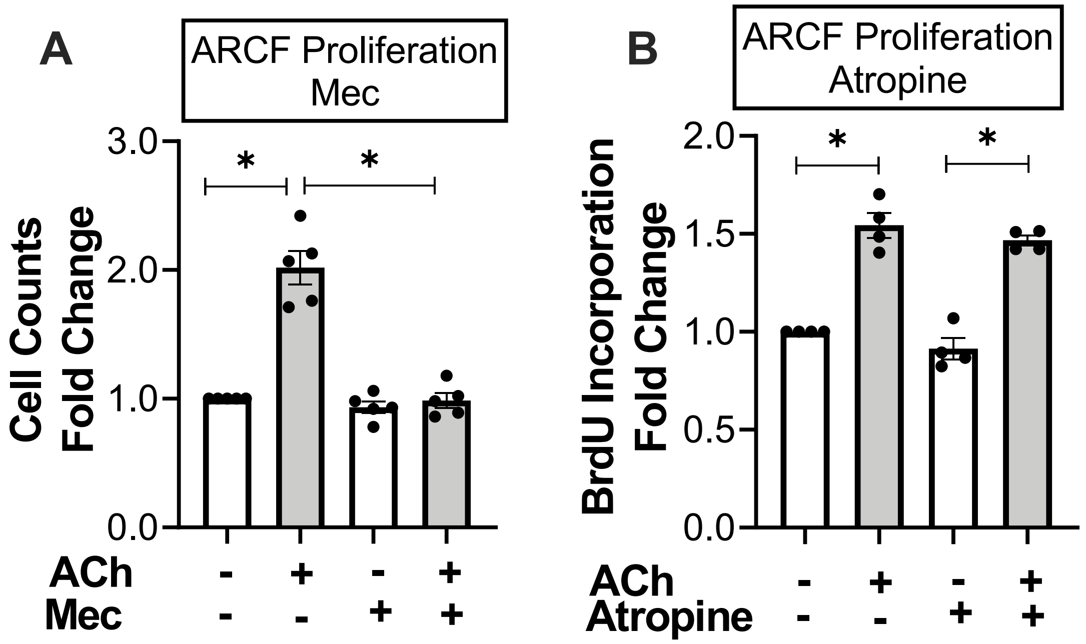

Figure S5. Effect of mecamylamine and atropine on Ach-induced proliferation of adult rat cardiac fibroblasts.
Experimental details similar to Figure 3C & 3E. Cell proliferation in adult rat CF upon ACh treatment in presence or absence of (**A**) mecamylamine (n=5 each) or (**B**) atropine (n=4 each). Mean ± SEM. Two-way ANOVA followed by Bonferroni comparison test. *$P < 0.05$. ARCF: adult rat CF, ACh: acetylcholine, Mec: mecamylamine, BrdU: Bromodeoxyuridine assay.

Figure S6

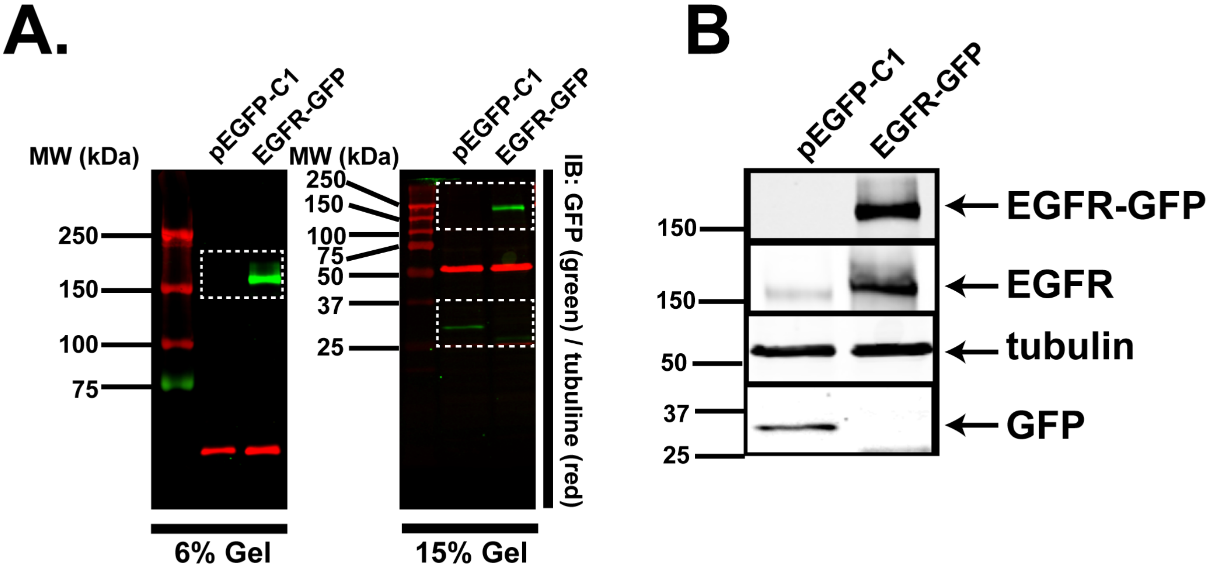

Figure S6. Generation of HEK293T cells stably overexpressing EGFR

(A) Representative fluorescent Western blots of EGFR-GFP detected by an infrared imaging system. Whole cell lysates were generated from HEK293T cells stably overexpressing EGFR-GFP and pEGFP-C1 vector (as a control). Immunoreactive bands corresponding to EGFR-GFP and GFP are shown in dotted squares. IB, immunoblot; MW, molecular weight. **(B)** Detection of EGFR in HEK293T cell lines stably overexpressing EGFR-GFP and pEGFP-C1 vector. Endogenous and exogenous EGFR were detected by antibodies against EGFR and GFP, respectively.

Figure S7

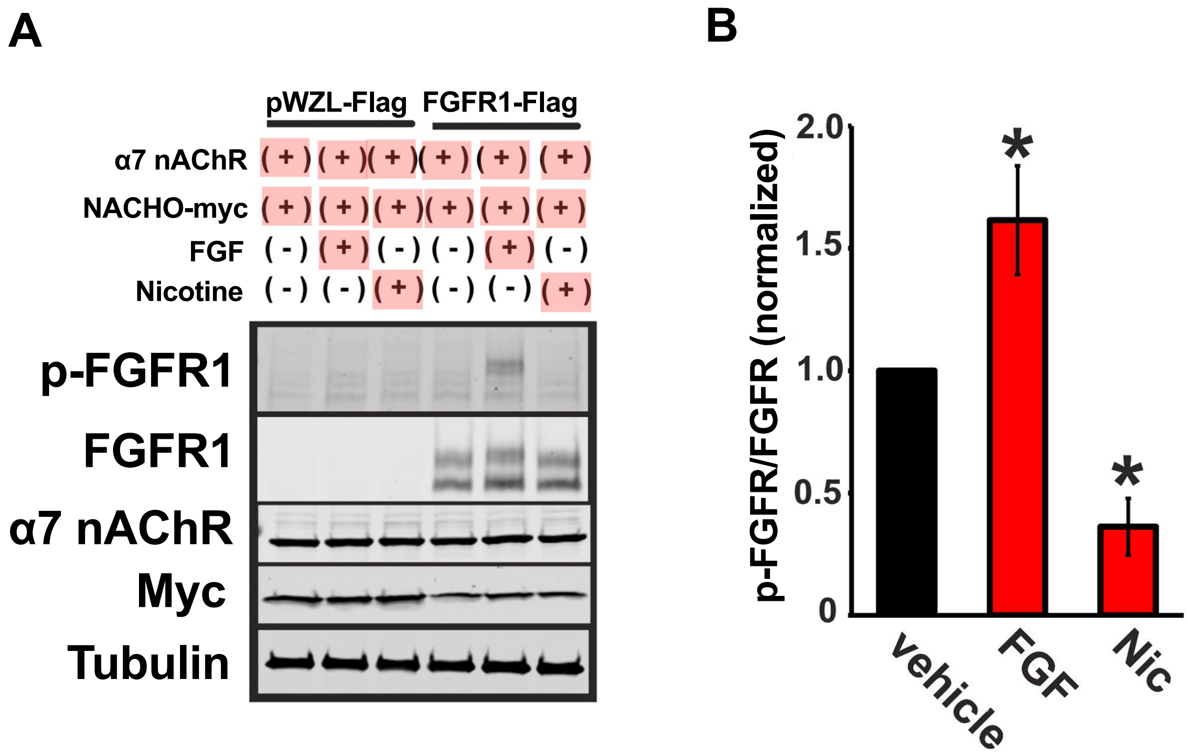

Figure S7. α7 AchR stimulation does not transactivates FGF receptors.
(A) Representative Western blots of phospho-FGFR and FGFR in HEK293T cells stably overexpressing FGFR1-flag. Cells stably expressing pWZL-flag were used as control. Cells were transiently transfected with α7 AchR and NACHO-Myc and stimulated with either with vehicle, 600 nM nicotine (Nic) and 1 ng/ml recombinant human FGF in the presence of 90 μg/ml heparin for 10 min. (B) Summary data of A. N=3, p*<0.05 compared to vehicle.

Figure S8

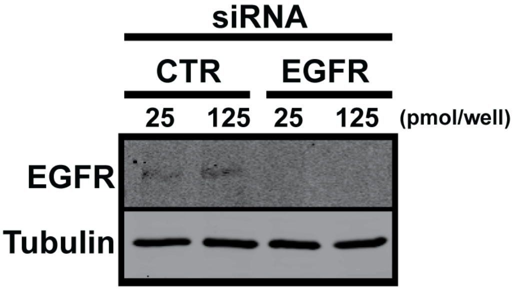

Figure S8. siRNA mediated molecular suppression of EGFR.

Adult rat cardiac fibroblasts were transfected with control or EGFR-specific siRNA at designated concentrations in 6-well plates, cultured for 72 hours, and harvested for protein lysates. Tubulin was used as loading control.

Figure S9

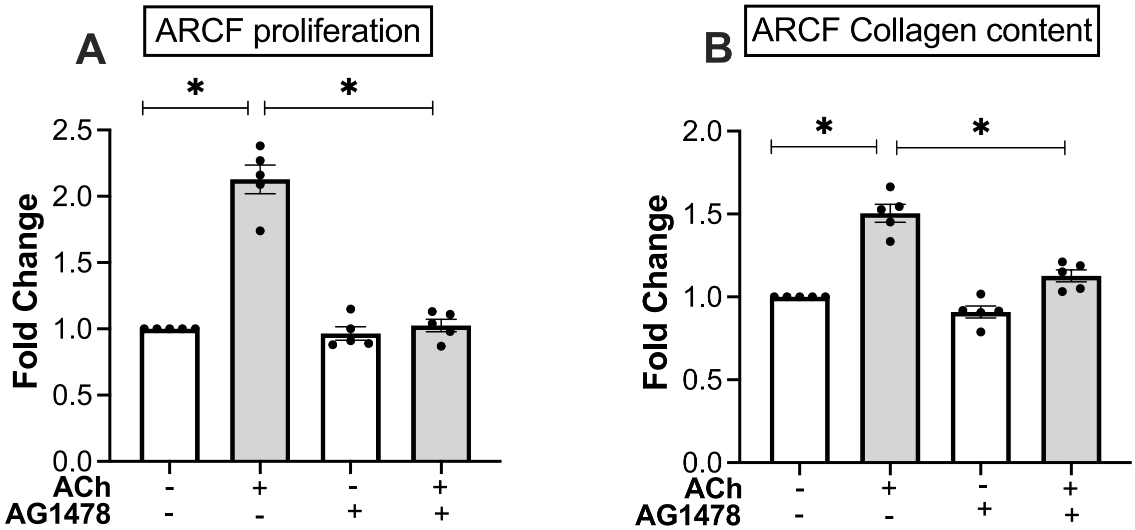

Figure S9. Effect of EGFR inhibition on ACh-induced ARCF proliferation and collagen production

Experimental design as in Figure 4I &J. (**A**) Cell counts (n=5 each) and (**B**) collagen content (n=5 each) of adult rat CF in response to ACh (10 nM) in the presence or absence of EGFR inhibitor AG1478 (50 μM). Mean ± SEM. Two-way ANOVA followed by Bonferroni comparison test. $*P < 0.05$. EGFR: epidermal growth factor receptor, AG1478: EGFR inhibitor, ARCF: adult rat CF, ACh: acetylcholine.

Figure S10

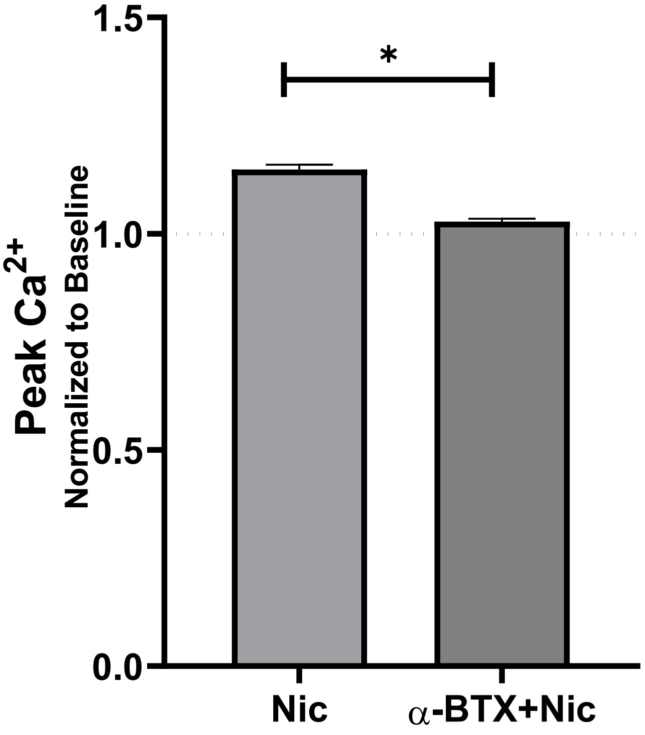

Figure S10. Elevation of intracellular Ca^{2+} levels in response to α7nAchR stimulation in adult rat cardiac fibroblasts.

Changes in $[Ca^{2+}]_c$ in response to nicotine stimulation were assessed by Ca^{2+}-sensitive dye Fluo-3 in live ARCFs in the presence (n=34) or absence (n=63) of α7nAchR-specific blocker α-BTX. Peak fluorescence value after stimulation was normalized by that before stimulation in each group. Two-tailed unpaired T-tests; *$P < 0.05$. α-BTX: alpha-bungarotoxin, Nic: Nicotine.

Figure S11

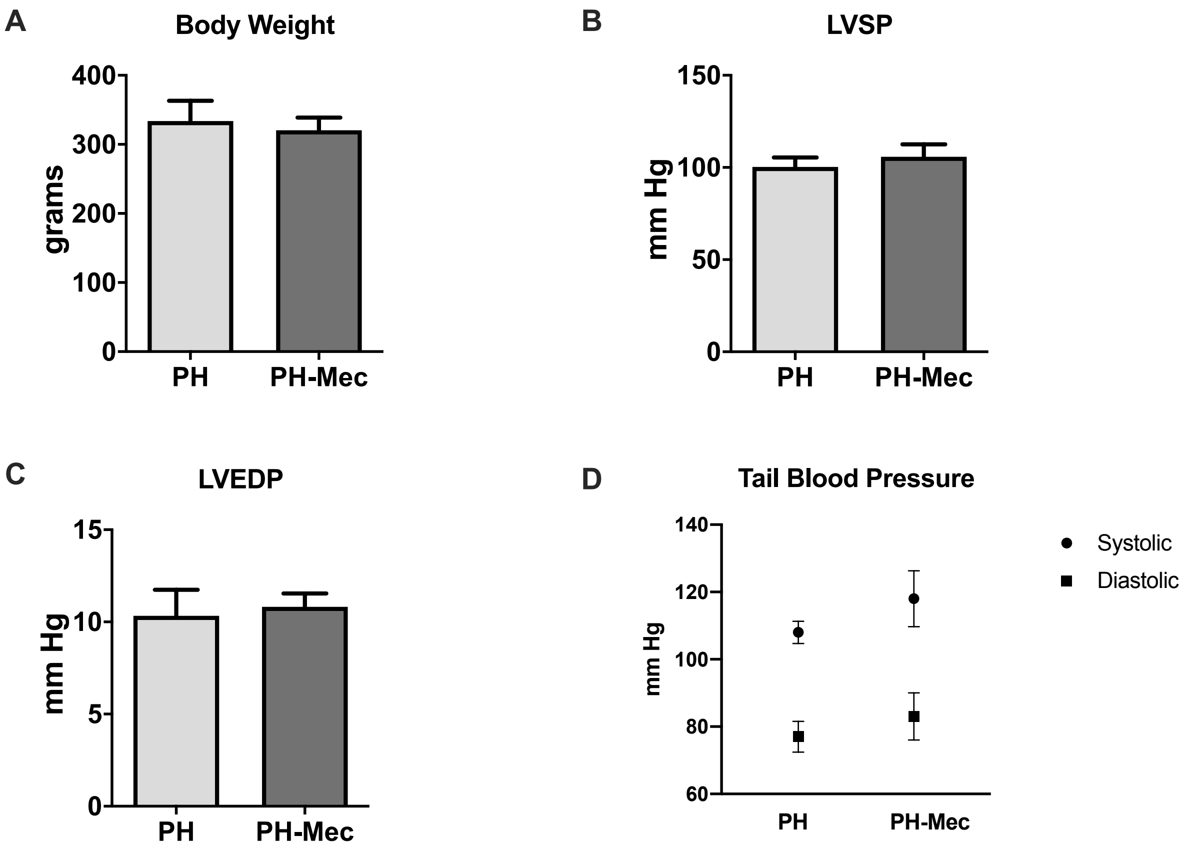

Figure S11. Effect of mecamylamine treatment on body weight, LV hemodynamics, and blood pressure.

Experimental design in Figure 5A. (**A**) Body weight, (B) LV systolic pressure, (C) LV end diastolic pressure and (D) systemic blood pressure measured using tail cuff in PH animals treated with either vehicle (PH-vehicle, n=9) or mecamylamine (PH-Mec, n=11). Mean ± SEM. Two-tailed *T*-tests were performed between PH and PH-Mec. PH: pulmonary hypertension, Mec: mecamylamine.

Figure S12

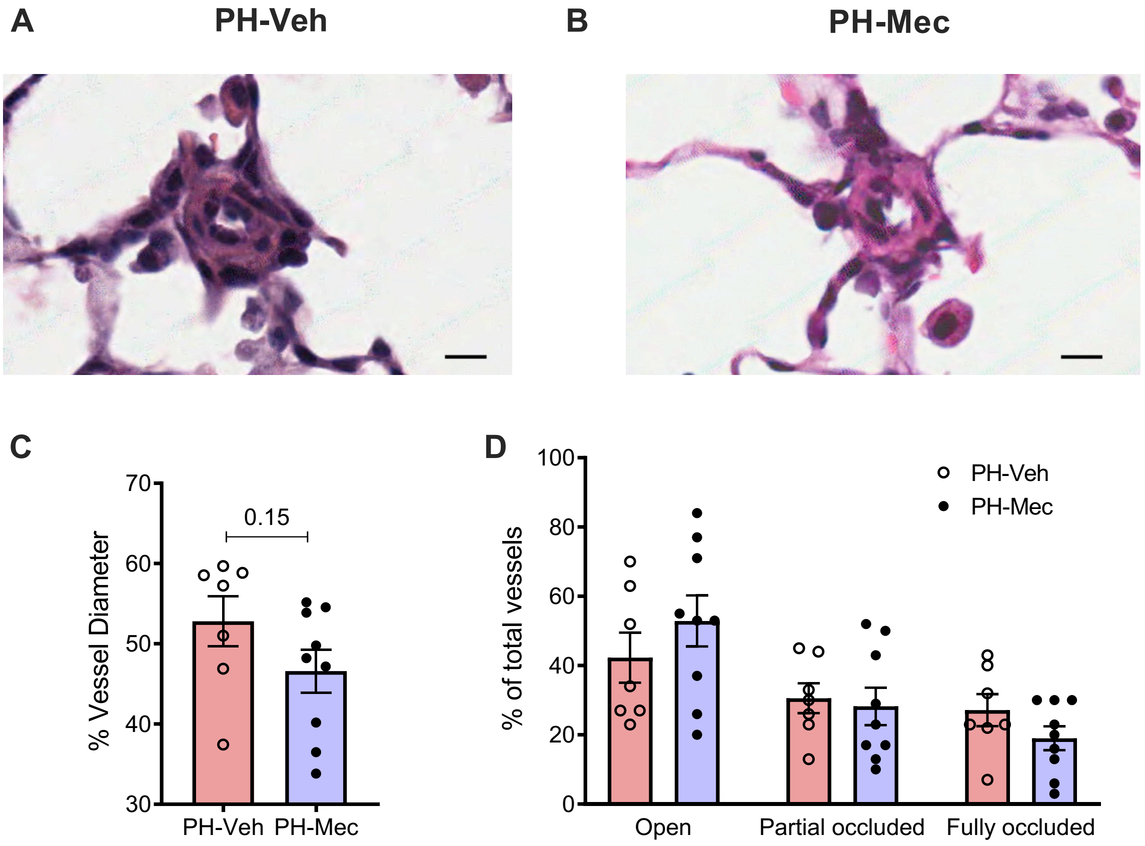

Figure S12. Effect of mecamylamine treatment on pulmonary vascular remodeling in PH. Experimental design in Figure 5A. Representative images of pulmonary arterioles (H&E, 40X magnification, scale bar 10 μm) for (**A**) vehicle treated PH (n=7) and (**B**) Mec treated (n=9) PH rats. (**C**) Medial wall thickness measured over 30 randomly distributed vessels normalized to vessel diameter. (**D**) Percentage of open and partially or fully occluded vascular lesions. Mean ± SEM. Two-tailed *T*-tests were performed between PH-Veh and PH-Mec. PH: pulmonary hypertension, Mec: mecamylamine.

Figure S13

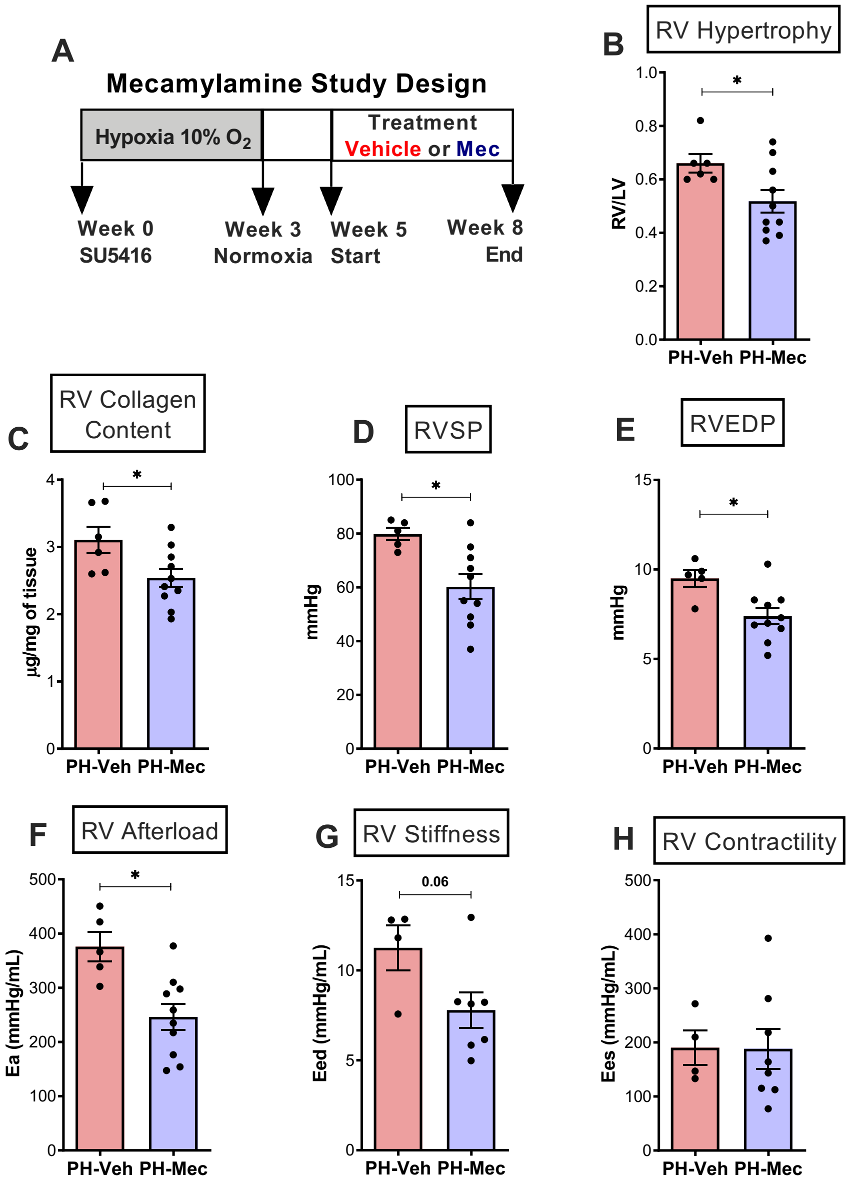

Figure S13: Treatment with nAChR blocker, mecamylamine, starting at 5 wks improves RV function in experimental pulmonary hypertension. (A) Schematic of experimental timeline: Rats were given a single bolus of SU5416, 20 mg/kg sq, and placed into hypobaric hypoxia chamber (10% FiO_2). After three weeks, rats were moved to room air. At the beginning of week five, rats were divided randomly and given vehicle or mecamylamine (20 mg/kg/d, ip) for three weeks. At the end of week 8, animals underwent terminal physiological measurements and sacrificed. **(B)** RV hypertrophy as measured by RV/LV. **(C)** RV collagen content as measured by Sircol assay. **(D/E)** RVSP and RVEDP as measured by PV catheter. **(F/G/H)** RV afterload, stiffness, contractility as measured from PV loops. N = 6-10 for B-E, 4-8 for F-H, *$P < 0.05$, unpaired t-test.

Figure S14

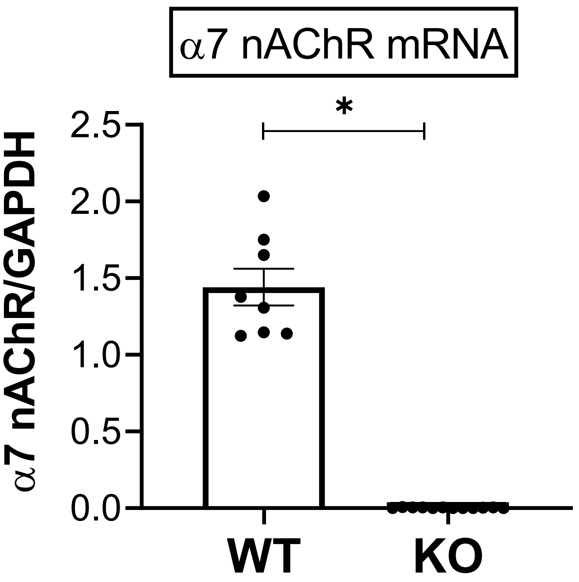

Figure S14. α7 nAChR mRNA expression in wild type and α7 nAChR knockout mice. mRNA expression confirming absence of α7 nAChR in RV of knockout mice (n=8/12). Mean ± SEM. Two-tailed *T*-test was performed between WT and KO mice; **P* < 0.05. nAChR: nicotinic acetylcholine receptor. WT: α7 nAChR wild type, KO: α7 nAChR knockout

Figure S15

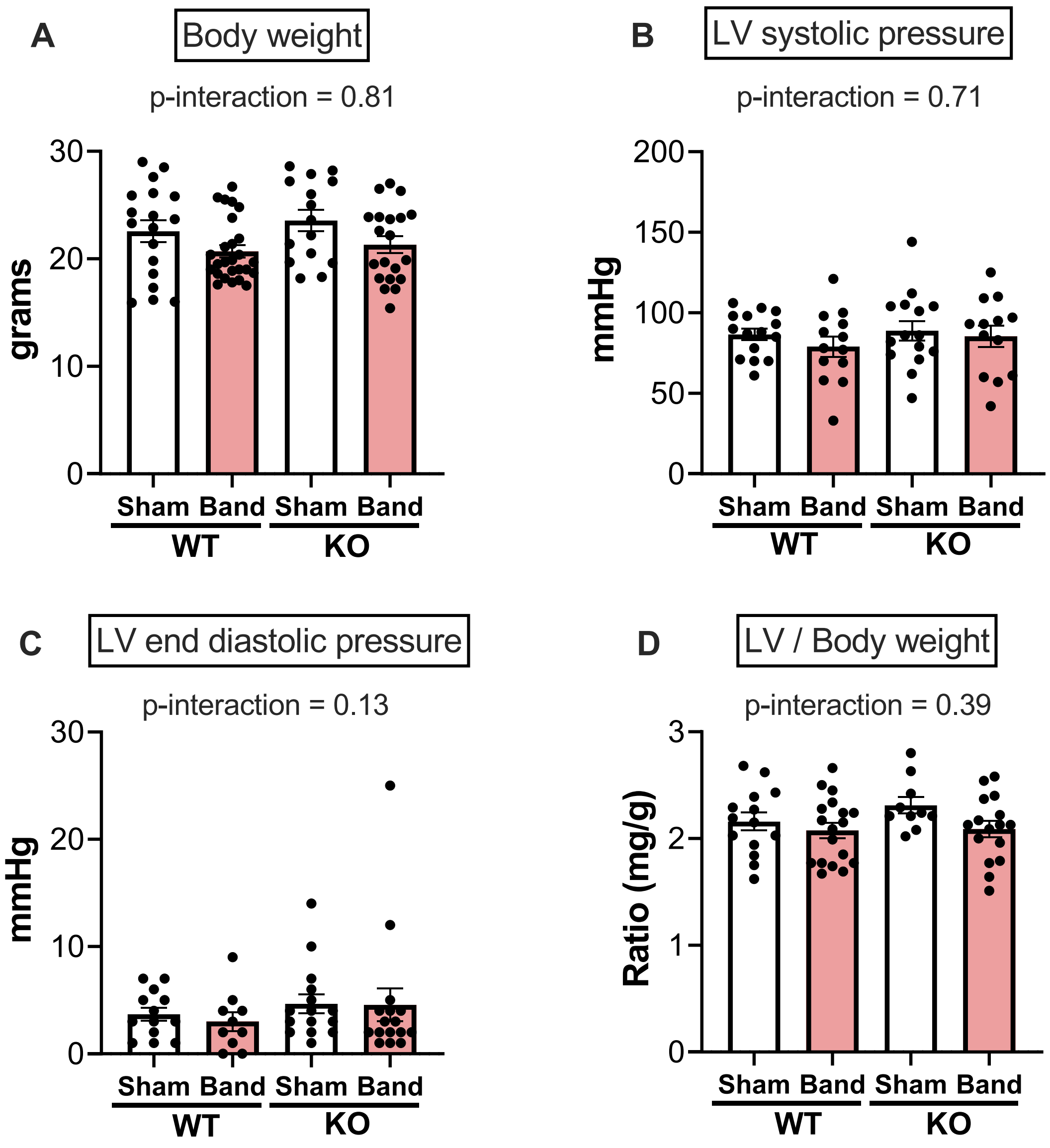

Figure S15. Body weight and left ventricular changes in wild-type and α7 nAChR knockout mice under RV pressure overload.

Experimental design as in Figure 6A. (**A**) Body weights of sham and PA banded, wild type (n=18/26) and knock-out (n=15/20) mice. (**B**) LV systolic (n=15/13 each) and (**C**) end diastolic pressures (wild type n=13/10; knock-out n=15/16). (**D**) LV weight divided by body weight after banding in either wild type (n=14/18) or knockout mice (n=10/16). Mean ± SEM. Two-way ANOVA followed by Bonferroni comparison test, not significant. WT: α7 nAChR+/+ wild type for alpha-7 nicotinic acetylcholine receptor, KO: α7 nAChR-/- knockout for alpha-7 nicotinic acetylcholine receptor, LV: left ventricle.
